# Gaps in the capacity of modern forage crops to adapt to the changing climate in northern Europe

**DOI:** 10.1007/s11027-016-9729-5

**Published:** 2016-12-07

**Authors:** Mäkinen Hanna, Kaseva Janne, Virkajärvi Perttu, Kahiluoto Helena

**Affiliations:** 1 School of Energy Systems, Sustainability Science, Lappeenranta University of Technology, Saimaankatu 11, 15140 Lahti, Finland; 2 Natural Resources Institute Finland, Latokartanonkaari 9, 00790 Helsinki, Finland; 3 Natural Resources Institute Finland, Tietotie 4, 31600 Jokioinen, Finland; 4 Natural Resources Institute Finland, Halolantie 31 A, 71750 Maaninka, Finland

**Keywords:** Adaptation, Genotype, Environment, Cultivar, Response diversity, Within-species response, Yield response

## Abstract

The within-species diversity in response to weather and the gaps in the response diversity in the modern set of forage crop cultivars were determined using an approach that assessed the adaptive capacity under global climate change. The annual dry matter (DM) yields were recorded in multi-location MTT (Maa- ja elintarviketalouden tutkimuskeskus) Agrifood Research Official Variety Trials in Finland for modern forage crop cultivars from 2000 to 2012, as a response to agroclimatic variables critical to yield based on the year-round weather data. The effect and interaction of cultivars and agroclimatic variables were analysed using mixed model. The relatively low adaptive capacity of timothy (*Phleum pratense* L.) and meadow fescue (*Festuca pratensis* Huds.) indicates that diversification of the breeding material is warranted, particularly for resistance to high temperatures during primary growth and to high temperature sum 7 days after the first cut. All red clover cultivars (*Trifolium pratense* L.) suffered from both low and high accumulation of warm winter temperatures. Except for the red clover cultivars, cold stress during winter and lack of warm winter temperatures consistently reduced the yields of all species and cultivars. All tall fescue (*Festuca arundinacea* Schreb.) cultivars suffered from low precipitation during the fall hardening period. Although the set of festulolium (*Festulolium pabulare*) cultivars was also sensitive to low precipitation during the fall, festulolium was a good example of enhanced capacity to adapt to climate change with high response diversity because the cultivar germplasm base was diversified. Foreign origin in a cultivar pool was apparently not sufficient or necessary to ensure added value for a diversity of responses to climate change. Similar analyses to those used in this study, applied as practical tools for breeders, farmers and public actors, are important to secure the adaptive capacity of crops worldwide under global climate change.

**Electronic supplementary material:**

The online version of this article (doi:10.1007/s11027-016-9729-5) contains supplementary material, which is available to authorized users.

## Introduction

Adaptation of crops to global climate change represents an urgent challenge for humanity. Although crops can be relatively well adapted to slow changes in the climate, the capacity to adapt to increased seasonal and inter-annual variation and to increased frequency of extremes may not be as high (Porter and Semenov 2005). Such potential vulnerability of agricultural production has created interest in building adaptive capacity and resilience (Smit and Wandel 2006; Lin 2011).

Dairy farming is the agricultural production line of highest economic significance in northern Europe, which is experiencing the most rapid climate change. Forage crops are the cornerstones in such production systems. In the future, forage production could benefit from several effects of climate change (Bindi and Olesen 2011; Graux et al. 2013; Rapacz et al. 2014), which include a longer physiologically effective growing season, a higher temperature sum and a higher concentration of CO_2_ (Porter et al. 2014) and better overwintering conditions that are predicted for some winter-sensitive species (Thorsen and Höglind 2010). As threats to the maintenance of sustainable forage production, increased frequency (Christidis et al. 2014) and duration of heat stress, droughts and heavy rain events are likely (Christensen and Christensen 2007; Ylhäisi et al. 2010; Ruosteenoja et al. 2011). Furthermore, climate change is expected to increase the risks of winter injury for most overwintering forages (Bélanger et al. 2002; Bertrand et al. 2003; Thorsen and Höglind 2010). A lack of full hardening during the fall and reduced snow cover may also expose crops to killing frosts, ice encasement and soil heaving (Bélanger et al. 2002; Rapacz et al. 2014).

In the context of climate change, ‘adaptive capacity is the ability of a system to adjust to climate change, including climate variability and extremes, to moderate potential damages, to take advantage of opportunities, or to cope with the consequences’ (IPCC 2001). Therefore, high adaptation is different from high adaptive capacity (Gallopín 2006; Smit and Wandel 2006), and a species or cultivar may be well adapted to the current environment but have little capacity to respond to variability and changes. We are unable to determine which cultivars will perform well in a future climate, but we may assume the requirements for capacity to adapt are likely to broaden (Coumou and Rahmstorf 2012; Chen and Tian 2016). Consequently, sufficient plasticity can hardly be bred within a single cultivar but rather within the entire cultivar pool. A key complement of the ability to respond is response diversity, meaning the diversity of responses within a functional group (Chapin et al. 1997; Elmqvist et al. 2003; Kahiluoto et al. 2014). Such diversity of responses to critical environmental change is likely to have a value that is proportional to its amount (Kahiluoto et al. 2014). Diversity of within-species responses in the available cultivar pool could increase yield stability under the current level of weather variability and ensure the potential for adaptation in the long-term as changes in the global climate proceed.

The aim of the study was to illustrate that assessments of response diversity can be used to identify gaps in the adaptive capacity to climate change. Specifically, the aim was to investigate whether within-species diversity in responses was apparent to changing and varying weather for the available set of modern forage crop cultivars as the most important crops grown in northern Europe: timothy (*Phleum pratense* L.), meadow fescue (*Festuca pratensis* Huds.), tall fescue (*Festuca arundinacea* Schreb.; syn. *Lolium arundinaceum* Schreb), tall fescue-type festulolium (*F. arundinacea* Schreb. × *Lolium multiflorum* Lam, i.e., *Festulolium pabulare*) that were backcrossed with *F. arundinacea* (DLF 2016) and red clover (*Trifolium pratense* L.). The annual forage crop dry matter (DM) yield (kg ha^−1^ year^−1^) was used throughout the study as the response variable to indicate adaptive capacity to agroclimatic variables critical to yield. The specific research questions were as follows:

1. Is there within-species diversity in responses to critical agroclimatic variables in the available set of modern forage crop cultivars?
2. To enhance the adaptive capacity, to which agroclimatic variables are the diversity of responses low?

## Materials and methods

### Variety trials

The Official Variety Trial results from MTT Agrifood Research Finland were used (Kangas et al. 2009). The Official Variety Trial data consisted of 8361 yield records for 126 cultivars from 16 different trial sites from 1980 to 2012. In this study, modern cultivars from 2000 to 2012 were selected from the following species: timothy, meadow fescue, tall fescue, festulolium and red clover. There were 1156 records of annual DM yields (kg DM ha^−1^ year^−1^) for 39 modern cultivars (Table 1). However, the entire data set from 1980 to 2012 was used in the analyses because every year the set of the tested cultivars changed slightly and thus long-term control cultivars improve the accuracy of DM yield estimates of modern cultivars. Annual DM yields (kg DM ha^−1^ year^−1^) were used as a response variable because DM yield can be used as a proxy for variation in food supply and farm income, and yield is also an indicator of ecosystem health (Rapport et al. 1998). Cultivars were of Finnish and foreign origin (Online resource 1), and each cultivar was associated with 20 to 70 observations from 11 different trial sites. All experiments at all sites were arranged as randomized complete block designs or incomplete block designs with three or four replicates. Plot size was 7–10 m × 1·25 m, depending on location and year. The experimental design and the management were similar to that on-farm, as described in more detail by Kangas et al. (2009) and Hakala et al. (2012).

**Table 1:** Experimental sites and the number of observations of modern cultivars (2000–2012) per site

Site	Latitude north	Longitude east	Timothy	Meadow fescue	Tall fescue	Festulolium	Red clover	All
Jokioinen	60° 49′	23° 30′	53 (13)	15 (7)	1 (1)	3 (3)	12 (3)	84 (27)
Piikkiö	60° 26′	22° 33′	18 (3)	12 (9)	7 (4)	15 (9)	9 (3)	61 (28)
Mietoinen	60° 38′	21° 55′	8 (5)	7 (5)	5 (5)	2 (2)	–	22 (17)
Kokemäki	61° 17′	22° 15′	11 (8)	6 (4)	4 (4)	5 (5)	–	26 (21)
Mikkeli	61° 40′	27° 10′	58 (19)	30 (18)	12 (9)	18 (15)	18 (6)	136 (67)
Maaninka	63° 09′	27° 19′	69 (16)	38 (19)	22 (13)	12 (9)	15 (6)	156 (63)
Laukaa	62° 19′	26° 19′	27 (9)	18 (9)	6 (6)	3 (3)	–	54 (27)
Ylistaro	62° 57′	22° 30′	76 (18)	34 (17)	12 (9)	12 (9)	18 (6)	152 (59)
Ruukki	64° 40′	25° 06′	81 (23)	40 (20)	12 (5)	6 (6)	18 (6)	157 (60)
Rovaniemi	66° 29′	25° 43′	69 (18)	36 (18)	15 (6)	6 (6)	14 (6)	140 (54)
Sotkamo	64° 01′	28° 22′	87 (19)	36 (18)	15 (6)	9 (6)	21 (9)	168 (58)
Total			557	272	111	91	125	1156

The numbers of trials per site and species are in parentheses

**Fig. 1:**
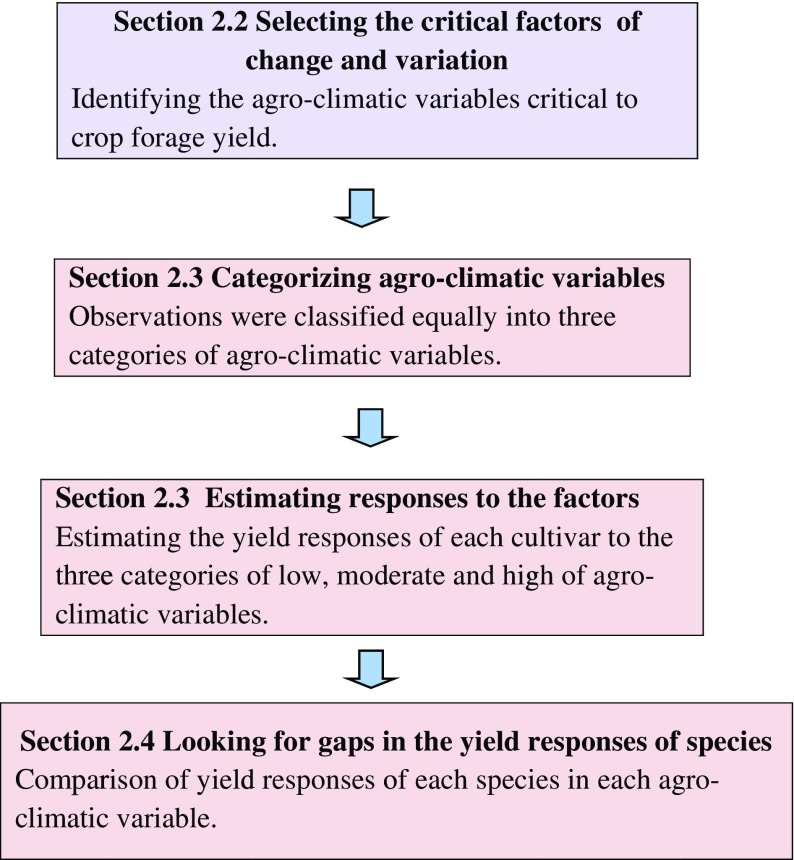
A flow diagram describing the steps of the analysis

The forage stands were productive 3 (to four) years after the establishment year and were harvested two to three times per year. The harvest dates varied among years and trials because of weather conditions and trial locations (e.g., latitude and soil type). A few harvest dates were missing (14 of 1409), which were estimated using a linear mixed model, relying on data for harvest dates of other cultivars at the same site and year. The effects of the site, year and species were considered in the model. The trials with missing data for the growing season were not included in the estimation of the variables. A flow diagram (Fig. 1) describes the steps of the following analysis.

### Selecting the critical factors of change and variation

The year-round daily weather data (1979–2012) from the Finnish Meteorological Institute for weather stations closest to the Official Variety Trials were used to assess the yield response to agroclimatic variables for each species and their cultivars separately. We identified agroclimatic variables critical for the forage yield performance in northern Europe using linear mixed models (Mäkinen et al. 2015); the yield responses were calculated for 35 pre-selected agroclimatic variables, and the agroclimatic variables were removed that, among other reasons, had a nonsignificant effect, minor effect or were missing too much information. In this study, these previously suggested critical agroclimatic variables (for details, see Mäkinen et al. 2015) were tested regarding the within-species diversity of a modern set of forage crop cultivars (Table 2).

**Table 2:** Agroclimatic variables and their medians for the three categories of values for the variables (low, moderate and high). Each category included one third of the observations

Period	Agroclimatic variable	Category Low median	Category Moderate median	Category High median
Fall hardening period (FH)	Length of fall hardening, days	39	64	99
FH started from the last day when the sum of daily difference between cold degree-days below 5 °C and degree-days above 5 °C was 0 after 1 August and continued to the last day of the first occurrence of minimum air temperatures ≤−10 °C	Accumulation of cold temperatures during FH <5 °C, degree-days	5.7	9.2	12.1
	Mean daily rainfall during FH, mm	1.44	2.03	2.67
Winter period (WP)	Mean daily accumulation of temperature >0 °C during WP, degree-days	0.56	0.77	1.03
WP started from the day following the end of FH and continued to the end of the day before the GP began, which started from the fifth day of the first 5-day spell when daily mean air temperature exceeded 5 °C	Accumulation of cold stress days with temperature <−15 °C, days	5	16	30
Growth period (GP)	Temperature sum >5 °C, degree-days	868	1040	1193
GP started from the fifth day of the first 5-day spell when daily mean air temperature exceeded 5 °C and ended with the last day of harvest	Mean daily temperature sum accumulation rate, degree-days	12.1	13.3	14.4
	Number of days with maximum temperature of 25 °C from GP start to 1st cut, days	0	1	5
	Number of days with maximum temperature of 25 °C from 1st cut to 2nd cut, days	1	5	12
	Accumulation of temperature sum 7 days after 1st cut, degree-days	103	121	141
	Accumulation of precipitation from GP start to 1st cut, mm	49.6	86.8	131.2
	Accumulation of precipitation from 1st cut to 2nd cut, mm	63.2	109.5	175.1
	Accumulation of precipitation 14 days after 1st cut, mm	11.3	29.7	57.2

The agroclimatic variables were preliminarily selected based on published literature (Bélanger et al. 2002; Volenec and Nelson 2007; Thorsen and Höglind 2010). The fall hardening period (FH), winter period (WP) and growth period (GP) were distinguished as suggested by Thorsen and Höglind (2010) and Bélanger et al. (2002). Regarding the FH and WP, the agroclimatic variables suggested by Thorsen and Höglind (2010) and Bélanger et al. (2002) were used. Different temperatures and precipitation rates were tested during the GP, based on the optimum temperatures for the cool season grasses suggested by Volenec and Nelson (2007). Because the development of forage crops is faster and characterized by different tiller traits during the primary growth stage, in comparison with the regrowth stage (Bélanger and McQueen 1998; Virkajärvi et al. 2012), some of the temperature and precipitation variables were tested separately for the first and the second harvest.

### Categorizing agroclimatic variables

Agroclimatic variables were analysed separately to investigate the gaps in the adaptive capacity of cultivars. Some agroclimatic variables were also strongly correlated leading to multicollinearity in regression analysis. The assumptions of linearity were also violated because the relations between the yield and the agroclimatic variables were nonlinear in most cases. Most importantly, we had to consider that the random effects of year, site and experiment contained most of the total variation; therefore, linear mixed models were used in data analyses.

Each agroclimatic variable was divided into three categories (low, moderate and high; Table 2) (Hakala et al. 2012). Trial observations were divided equally into the three categories to ensure the reliability of yield estimates. For example, the number of cold stress days during the winter period (W-STRESS) was in the low category for 0–10 days, in the moderate category for 11–21 days and in the high category for more than 21 days. We used a minimum requirement of 20 observations per cultivar together with possible missing values in a random agro-climatic variable. More uneven division of observations would have led to categories with few observations only for some agroclimatic variables.

### Estimating responses to the factors

The main effects and interaction of cultivars (G = genotype) and categorical agroclimatic variable (E = environment) were analysed for each species separately using the following mixed model:

$$ {\mathrm{y}}_{ijklm}=\mu +{\mathrm{cultivar}}_i+{\mathrm{category}}_j+\mathrm{cultivar}\times {\mathrm{category}}_{ij}+\mathrm{experimental}\ \mathrm{site}\times \mathrm{year}\times \mathrm{trial}{\left(\mathrm{category}\right)}_{klmj}+{\varepsilon}_{ijklm} $$

where *y*_*ijklm*_ is the observed yield (annual DM yield), *μ* is the intercept, cultivar _*i*_ is the average yield level of the *i*th cultivar, category_*j*_ is the average yield level at the *j*th level of categorized environment (*j* = 1, 2, 3) and cultivar × category_*ij*_ is the cultivar-by-environment interaction. All of the above effects are fixed in the model. Experimental site × year × trial(category)_*jklm*_ is the random effect of the *k*th experimental site, *l*th year and *m*th trial within the *j*th category and *ε*_*ijklm*_ is a normally distributed residual error.

### Looking for gaps in the yield responses of species

**Fig. 2.**
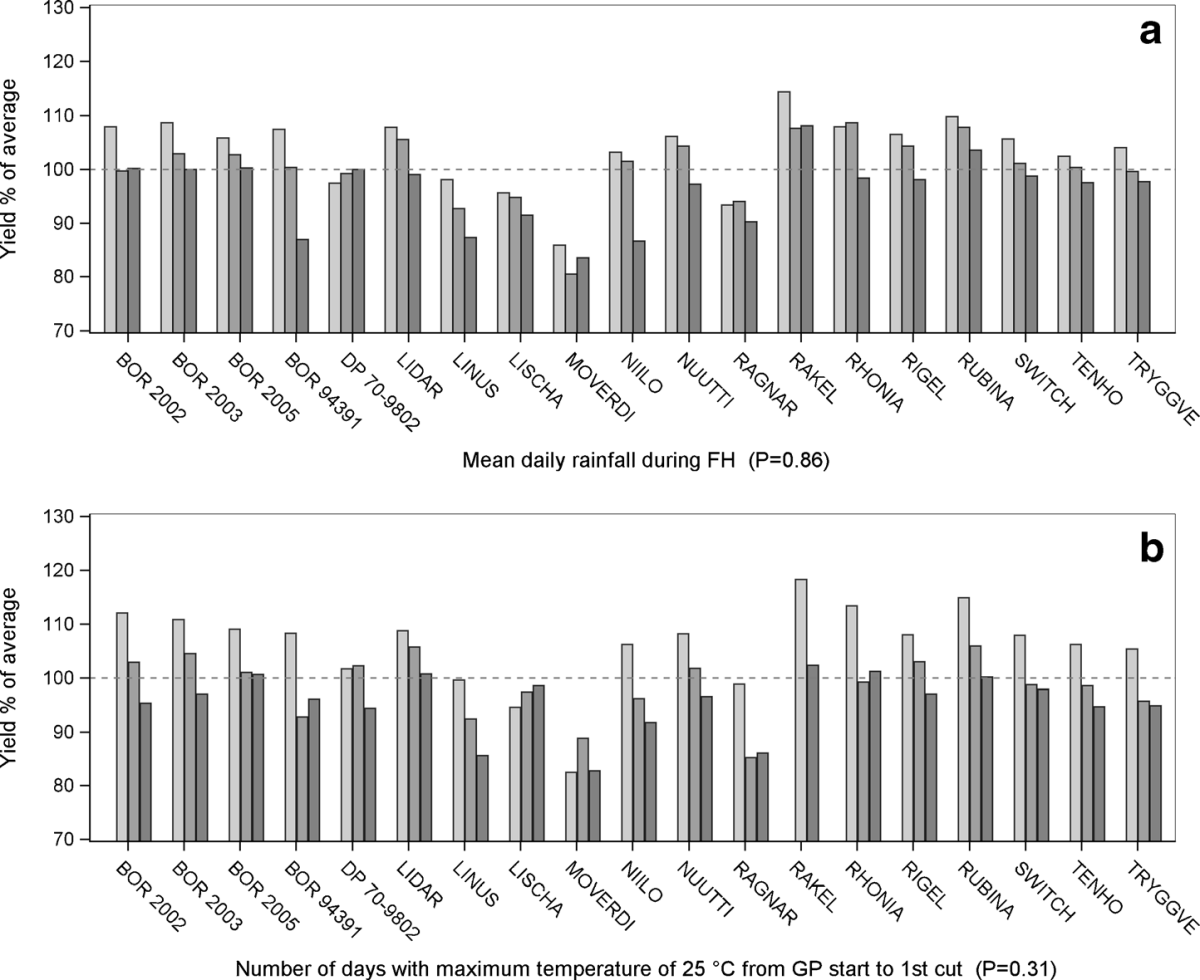
Yield responses of timothy cultivars (G × E). The *dashed line* indicates 100 % of the average yields of the cultivars for 2000–2012. *P* values are for the statistical significance for the interaction between the cultivar and the weather variable. The columns are as follows: low , moderate  and high  accumulation of **a** mean daily rainfall during the fall hardening period and **b** number of days with the maximum temperature of 25 °C from the start of the growth period to the first cut. For further details regarding the low, moderate and high categories, see Table 2

**Fig. 3:**
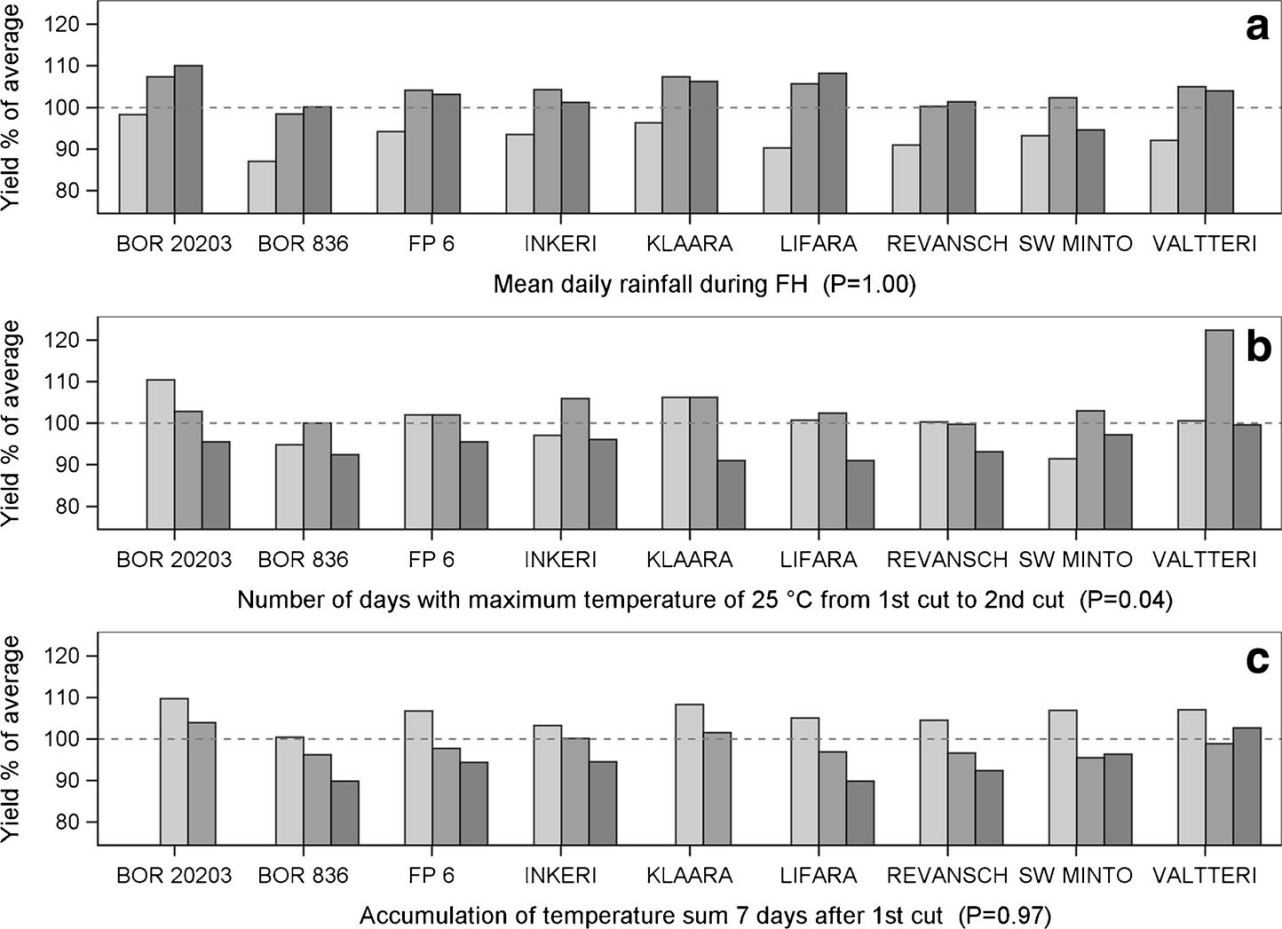
Yield responses of meadow fescue cultivars (G × E). The *dashed line* indicates 100 % of the average yields of the cultivars for 2000–2012. *P* values are for the statistical significance for the interaction between the cultivar and the weather variable. The columns are as follows: low , moderate  and high  accumulation of **a** mean daily rainfall during FH, **b** number of days with the maximum temperature of 25 °C from the first cut to the second cut and **c** temperature sum 7 days after the first cut. For further details regarding the low, moderate and high categories, see Table 2

**Fig. 4:**
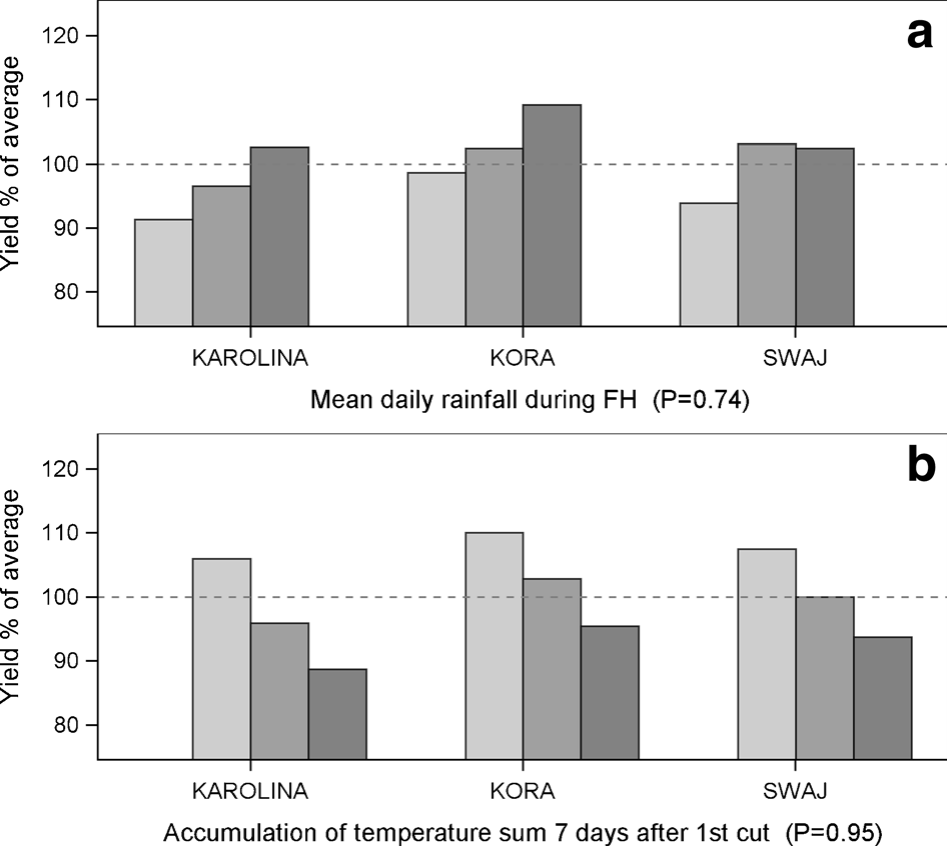
Yield responses of tall fescue cultivars (G × E). The *dashed line* indicates 100 % of the average yields of the cultivars for 2000–2012. *P* values are for the statistical significance for the interaction between the cultivar and the weather variable. The columns are as follows: low , moderate  and high  accumulation of **a** mean daily rainfall during FH and **b** temperature sum 7 days after the first cut. For further details regarding the low, moderate and high categories, see Table 2

**Fig. 5:**
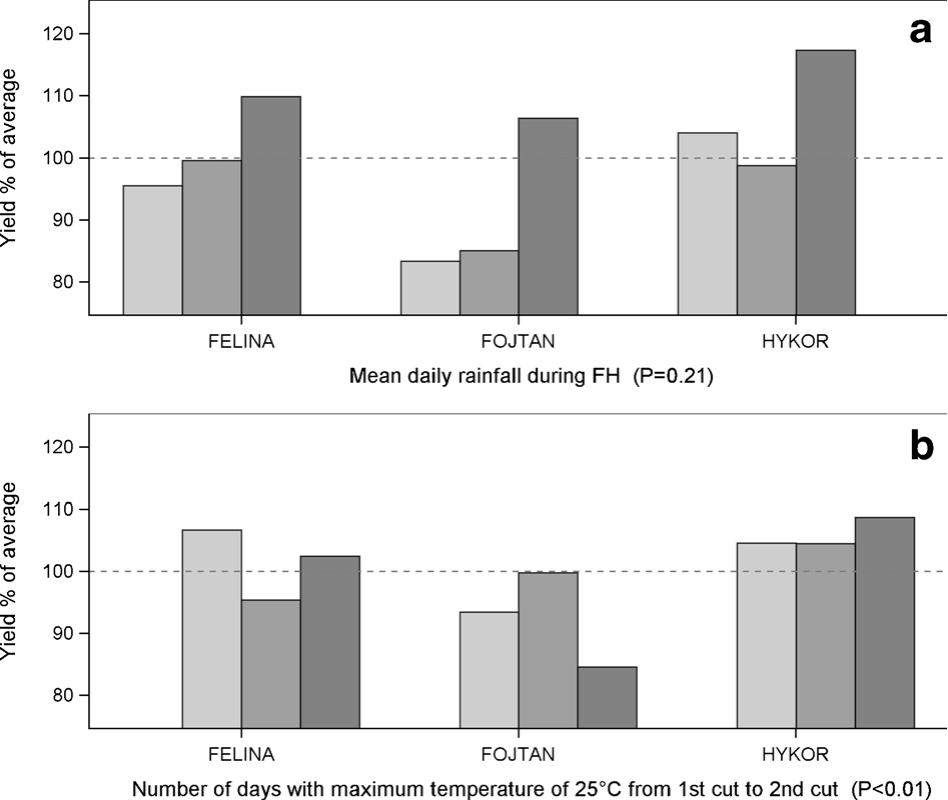
Yield responses of festulolium cultivars (G × E). The *dashed line* indicates 100 % of the average yields of the cultivars for 2000–2012. *P* values are for the statistical significance for the interaction between the cultivar and the weather variable. The columns are as follow: low , moderate  and high  accumulation of **a** mean daily rainfall during the FH and **b** number of days with the maximum temperature of 25 °C from the first cut to the second cut. For further details regarding the low, moderate and high categories, see Table 2

**Fig. 6:**
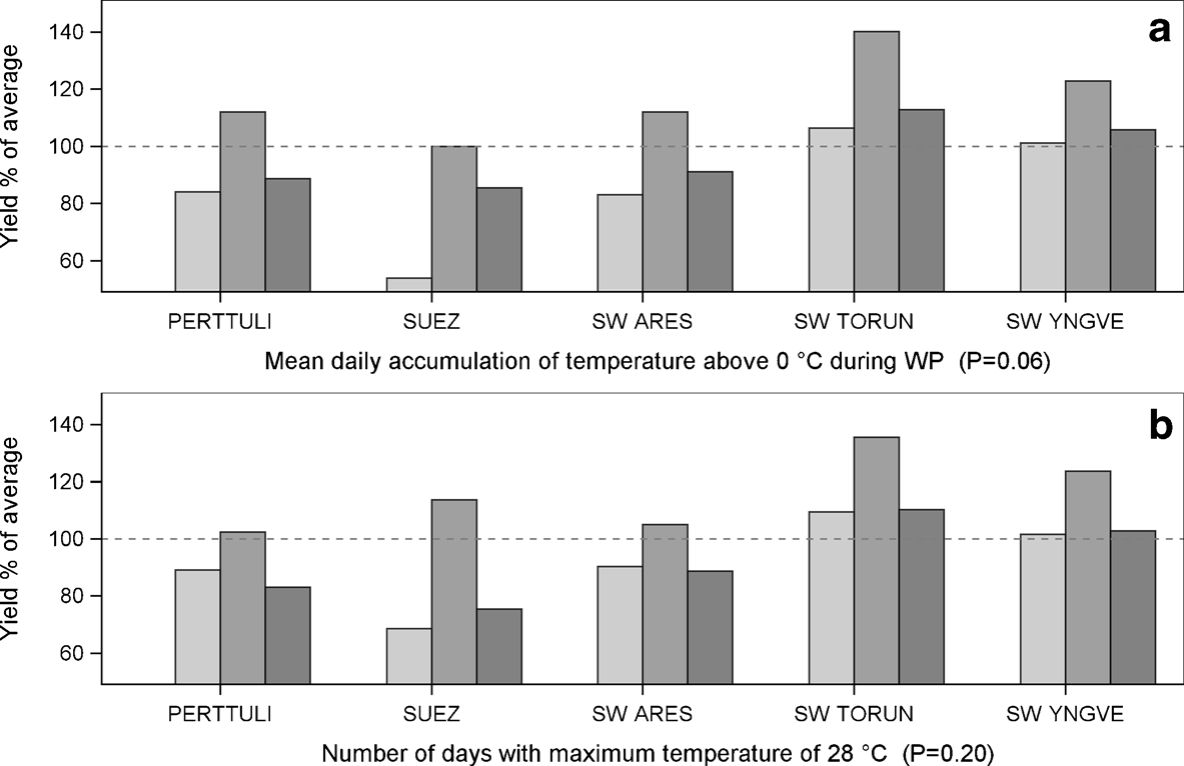
Yield responses of red clover cultivars (G × E). The *dashed line* indicates 100 % of the average yields of the cultivars for 2000–2012. *P* values are for the statistical significance for the interaction between the cultivar and the weather variable. The columns are as follows: low , moderate  and high  accumulation of **a** mean daily accumulation of temperature above 0 °C during the WP and **b** number of days with the maximum temperature of 28 °C. For further details regarding the low, moderate and high categories, see Table 2

*P* values of statistical significance for G, E and G × E were calculated. Although a clear response was indicated for cases in which E was statistically significant but G × E was not, the response diversity within the cultivars of a species was low in these cases, and therefore, a gap in adaptive capacity was identified. The results are illustrated in figures regarding those agroclimatic variables with the most diversity in responses and those with the least (Figs. 2, 3, 4, 5 and 6 and Table 4).

Statistical analyses were performed using the MIXED procedure in the SAS statistical software package (version 9.3, SAS Institute Inc., Cary, NC, USA).

## Results

### Timothy

Modern timothy cultivars showed no differences in yield responses to the agroclimatic variables (Table 3). The cultivars suffered from high precipitation during FH (Fig. 2a), and a long FH decreased the yields by more than 5 % in all cultivars (Table 4).

**Table 3:** Variation in forage crop yield responses to agroclimatic variables among cultivars

Period	Agroclimatic variable	Species									
		Timothy		Meadow fescue		Festulolium		Tall fescue		Red clover	
		max diff	*P* value	max diff	*P* value	max diff	*P* value	max diff	*P* value	max diff	*P* value
FH (fall hardening period)	Length of FH	−1921	0.96 (**)	763	0.65 (0.67)	1006	(0.71)**	1001	0.24 (0.22)	1128	(0.64)**
	Accumulation of cold temperatures during FH below 5 °C	977	0.90 (0.87)	−1028	0.62 (***)	−1560	(0.11)**	−1088	0.80 (**)	1239	0.37 (0.62)
	Mean daily rainfall during FH	−1904	0.86 (*)	1536	1.00 (***)	2155	0.21 (****)	1142	0.74 (**)	1011	0.27 (0.76)
WP (winter period)	Mean daily accumulation of temperature above 0 °C during WP	2100	0.53 (***)	2638	0.43 (****)	2426	0.17 (****)	2584	0.27 (****)	2114	–* (***)
	Cold stress days with temperature below −15 °C during WP	−1287	0.25 (****)	−2159	0.29 (****)	−1997	0.15 (****)	−1874	0.31 (****)	1145	0.40 (0.21)
GP (growth period)	Temperature sum	1870	0.81 (****)	−1067	0.82 (**)	3367	–** (****)	2203	–*** (****)	2481	0.13 (****)
	Mean daily temperature sum accumulation rate	1702	0.47 (**)	−1026	0.15 (*)	579	0.15 (0.13)	589	0.11 (0.91)	2747	0.60 (****)
	Number of days with maximum temperature of 28 °C	2756	0.97 (***)	1236	0.51 (0.42)	1197	(0.39)***	755	0.04 (0.33)	459	0.20 (****)
	Number of days with maximum temperature of 25 °C from GP start to 1st cut	−1503	0.31 (***)	−1355	0.14 (0.22)	946	–** (***)	−628	0.30 (0.23)	877	0.30 (0.50)
	Number of days with maximum temperature of 25 °C from 1st cut to 2nd cut	1554	0.50 (**)	−1354	–** (**)	−831	(0.72)***	−533	0.17 (0.94)	2542	–* (**)
	Accumulation of temperature sum 7 days after 1st cut	−3141	(0.39)*	−1355	0.97 (**)	−1770	0.12 (***)	−1750	0.95 (****)	−1029	(0.43)*
	Accumulation of precipitation from GP start to 1st cut	−967	0.63 (0.97)	757	0.97 (0.65)	−3049	–**** (***)	291	0.31 (0.77)	655	0.11 (0.42)
	Accumulation of precipitation from 1st cut to 2nd cut	−1609	0.71 (0.40)	895	0.36 (0.27)	−1668	–**** (0.68)	−592	0.39 (0.89)	1253	0.26 (0.45)
	Accumulation of precipitation 14 days after 1st cut	−1516	0.51 (0.74)	1229	0.49 (0.21)	756	0.11 (0.32)	350	0.94 (0.56)	1013	0.32 (0.16)

The difference between the largest and the smallest yield response among the cultivars of each species to each agroclimatic variable (max diff) and the statistical significance of the interaction (G × E) and of the effect of each agroclimatic variable (E) on yield of the species (*P* value) are shown

*P = 0.10, **P = 0.05, ***P = 0.01 and ****0.001 levels of probability for E in parentheses

**Table 4:** Effects of selected agroclimatic variables on the average yield of species

Species	Agroclimatic variable	Category low % of the average yield	Category high % of the average yield
Timothy	Mean daily rainfall during FH, mm	104	96
	Length of fall hardening period, days	105	95
	Number of days with maximum temperature of 25 °C from GP start to 1st cut, days	106	96
Meadow fescue	Mean daily rainfall during FH, mm	93	103
	Accumulation of cold stress days with temperature <−15 °C, days	104	89
	Mean daily accumulation of temperature >0 °C during WP, degree-days	93	111
	Number of days with maximum temperature of 25 °C from 1st cut to 2nd cut, days	100	95
	Accumulation of temperature sum 7 days after 1st cut, degree-days	104	94
Tall fescue	Accumulation of cold temperatures during FH <5 °C, degree-days	105	96
	Mean daily rainfall during FH, mm	95	105
	Accumulation of cold stress days with temperature <−15 °C, days	105	89
	Mean daily accumulation of temperature >0 °C during WP, degree-days	91	113
	Accumulation of temperature sum 7 days after 1st cut, degree-days	108	93
Festulolium	Mean daily rainfall during FH, mm	94	111
	Accumulation of cold stress days with temperature <−15 °C, days	104	87
	Mean daily accumulation of temperature >0 °C during WP, degree-days	92	114
	Accumulation of temperature sum 7 days after 1st cut, degree-days	106	99
Red clover	Number of days with maximum temperature of 28 °C, days	92	92
	Temperature sum >5 °C, degree-days	84	106
	Mean daily temperature sum accumulation rate, degree-days	80	109

In the growth period, all cultivars were sensitive to the high accumulation of days with temperature of 25 °C during the primary growth (Fig. 2b) (see Online resource 2).

### Meadow fescue

Meadow fescue cultivars responded differently to one of the 14 agroclimatic variables tested (Table 3). All cultivars showed sensitivity to low precipitation during the fall hardening period (Fig. 3a). Additionally, the occurrence of a high number of cold winter temperatures (>−15 °C) consistently reduced the yields by 10–25 %. A high accumulation of warm winter temperatures (above 0 °C) increased the yields nearly 20 % compared with those of the lowest temperature accumulation category (Table 4).

Regarding the growth period, diversity occurred in the yield responses to a high number of days with the maximum temperature of 25 °C during the regrowth stage (Fig. 3b); in most cases, the temperature stress led to marked yield reduction, but SW Minto and Valtteri cultivars yielded well under the moderate and high rates of temperature accumulation. A high temperature sum 7 days after the first cut reduced the yields of all cultivars (Fig. 3c) (see Online resource 3).

### Tall fescue

Tall fescue cultivars showed diversity in yield responses to two of the 14 agroclimatic variables (Table 3). During fall hardening, an increase in accumulation of cold temperatures <5 °C consistently reduced the yields, and in the lowest precipitation category, yield penalties occurred in all tall fescue cultivars (Fig. 4a). All tall fescue cultivars were apparently sensitive also to harsh winter conditions: yield reductions of approximately 15 % were found during winters characterized with a high number of days causing cold stress (−15 °C; Table 4). A high accumulation of warm winter temperatures (above 0 °C) increased the yields by approximately 20 % compared with those of the lowest accumulation category.

In the growth period, tall fescue cultivars differed in response to the temperature sum accumulation and to the number of days with the maximum temperature of 28 °C. However, a high temperature sum 7 days after the first cut was not tolerated well by any of the cultivars (Fig. 4b) (see Online resource 4).

### Festulolium

Festulolium cultivars showed differences in yield responses to eight of the 14 agroclimatic variables (Table 3). All cultivars were apparently sensitive to a low or moderate rate of precipitation during the fall hardening period (Fig. 5a). All festulolium cultivars were sensitive to the high accumulation of cold temperatures (−15 °C) during the winter period (10–21 % yield reduction). By contrast, a high accumulation of temperatures above 0 °C during the winter period increased yields markedly (Table 4).

During the growth period, the cultivars differed in their yield responses to the temperature sum. Cultivars differed in their responses to the number of days with the maximum temperature of 25 °C in the primary and regrowth stages (Fig. 5b). Cultivars also differed in their responses to very high temperature stress (28 °C) during the growth period. In most cases, the accumulation of a high temperature sum 7 days after the first cut led to a yield reduction. Cultivars responded differently to the accumulation of precipitation before the first cut (see Online resource 5).

### Red clover

The modern red clover cultivars showed differences in yield responses to one of the 14 agroclimatic variables and a tendency in responses to three of the 14 (Table 3). Although red clover cultivars were sensitive to winter temperatures that differed from the moderate accumulation of temperatures above 0 °C, there was a tendency towards differences in yield responses (Fig. 6a).

The highest (and the lowest) number of days of the very high temperature stress (+28 °C) led consistently to a yield reduction (Fig. 6b). The low temperature accumulation rate or the temperature sum led to marked yield reductions (Table 4) (see Online resource 6).

## Discussion

### Sensitivity of forage crop cultivars to climate change

We used the annual forage crop DM yield (kg ha^−1^ year^−1^) to indicate the response diversity as a proxy to adaptive capacity to weather variability throughout the study. Although the DM yield of the first cut is typically more sensitive than the annual harvest to changes in the fall hardening and winter related variables (Kangas et al. 2009), the annual DM yield is by far the most important variable for forage production because it is linked to farm fodder and food supply and farm income.

The length of the hardening period is associated with better winter survival of grasses (Bélanger et al. 2002). According to Bélanger et al. (2002), the mean length of fall hardening in eastern Canada is approximately 24 days, whereas in this study, the mean length of lowest category of the fall hardening period was 39 days (Table 2). This seems sufficiently long for the tested forage crop cultivars, since it did not result in marked damage. If precipitation during the fall increases as projected (Ruosteenoja et al. 2011), excessive soil moisture is likely to hamper the hardening (Paquin and Mehuys 1980), as indicated by our findings regarding yield penalties in the timothy cultivars, whereas other forage crop cultivars succeeded well under such conditions. In this regard, diversity in the breeding material of timothy is required to complement the new drainage measures.

According to our results, warm winter temperatures increased the yields of timothy, meadow fescue, festulolium and tall fescue cultivars, as also reported by Thorsen and Höglind (2010), wherein the occurrence of ice cover is rarely reported. Höglind et al. (2010) found that timothy cv. Grindstad was more sensitive to frost than cv. Engmo, but we did not establish differences among the timothy cultivars in responses to those tested winter weather variables. All modern red clover cultivars succeeded well under the moderate accumulation of warm winter temperatures. The sensitivity of red clover cultivars to the high accumulation of warm winter temperatures might be explained by dehardening and consequent winter damage (Bélanger et al. 2002). On the other hand, the sensitivity of the cultivars to the low accumulation of such warm winter temperatures and associated long-standing snow cover might be explained by the promotion of diseases such as clover rot (*Sclerotinia trifoliorum*) (Ylimäki 1967).

The expected elevated temperatures during the growing season combined with long days may disturb growth and result in overly rapid crop development in the future. Temperature stress reduces the growth and chlorophyll content and causes injuries to cell membranes (Heide 1982; Wang et al. 2009). Temperatures below 21 °C, particularly at approximately 17 °C, are suggested for the optimal growth of timothy (Smith 1972; Bertrand et al. 2008), which explained the sensitivity of timothy cultivars to temperature stress observed in our study. Unlike our findings for DM yields of timothy cultivars under field conditions, significant differences are reported for temperature tolerance in timothy cultivars in growth chamber assays (Heide 1982). For example, high temperatures apparently more easily inhibit the flowering of cultivars of Scandinavian origin than that of British or American cultivars (Cooper 1958). Whereas temperature stress was harmful for most of the tested meadow fescue cultivars, the cultivar Valtteri, which is of Finnish origin, performed better under hot conditions. Regarding tall fescue, studies show great variability among the cultivars in responses to temperature stress (Wang et al. 2009; Sun et al. 2015), such as we found for both tall fescue and festulolium. Red clover cultivars, as reported earlier (Fagerberg 1988), were not sensitive to the occurrence of days with the maximum temperature of 25 °C. However, we did show a marked yield reduction in the red clover cultivars when exposed to a high number of days with the maximum temperature of 28 °C. A high temperature sum 7 days after the first cut consistently reduced yields in all of the meadow fescue and tall fescue cultivars tested.

Climate change is expected to change patterns of precipitation, and in particular, early season droughts may limit the yield potential in Finland (Trnka et al. 2011). Although timothy has high root biomass (Bolinder et al. 2002), it has low tolerance for drought (Molyneux and Davies 1983; Wilman et al. 1998). However, no significant yield penalties for the timothy cultivars were shown for conditions of low precipitation in the primary growth and regrowth stages. The high soil water reserves in spring due to melting snow might have contributed to these results. The persistence of most of the tested fescue species to summer droughts was previously reported (Humphreys et al. 1997). A variation in root systems among genotypes, which has been reported, e.g. in a perennial ryegrass (Crush et al. 2007), might have affected our findings for festulolium, because yield responses of festulolium cultivars differed greatly according to different precipitation rates during the primary growth stage. We did not find any marked effect of precipitation-related weather variables on tall fescue and meadow fescue yields or on differences among the responses of cultivars, although Huang and Gao (1999) and Assuero et al. (2002) previously reported high levels of variation among tall fescue cultivars. All red clover cultivars suffered from dry conditions during regrowth, as also reported by Mela (2008) and Fagerberg (1988).

### Diversity of responses in the modern set of forage crop cultivars

Overall, when compared with barley cultivars (Hakala et al. 2012), the modern forage crop cultivars responded more similarly to the tested agroclimatic variables. The high diversity observed in the responses of festulolium cultivars was likely due to the diverse genetic base, because the festulolium cultivars were synthetic *F. arundinacea* × *L. multiflorum* hybrids (DLF 2016). By contrast, the similarity of responses observed for the timothy cultivars was likely due to the cross-pollinating that occurs with timothy. According to Tanhuanpää and Manninen (2012), a lack of differentiation in Nordic timothy is likely due to the substantial gene flow between cultivars and natural populations, which prevents geographical differentiation. However, they concluded that the timothy material apparently has sufficient diversity for breeding purposes (Tanhuanpää and Manninen 2012), although genetic variation for stress adaptation was not considered. In the set of meadow fescue cultivars from Nordic countries, most of the genetic variation occurs within populations rather than among different cultivars (Fjellheim and Rognli 2005), which explained our findings for meadow fescue. Considerably low levels of genetic variation are found for meadow fescue cultivars compared with perennial ryegrass or cocksfoot cultivars (Kölliker et al. 1999), and such a limited genetic variability likely contributed to the decline in the use of meadow fescue for grass production (Kölliker et al. 1999). Although we found significant differences in yield responses of the modern cultivars to some weather variables, we assume that variation will likely increase when the breeding includes a wider range of red clover (e.g. Helgadóttir 1996; Halling et al. 2004) and tall fescue (e.g. Mian et al. 2002; Majidi et al. 2009) cultivars.

### Towards enhanced adaptive capacity of forage crops

Effective adaptation is likely not possible without breeding programs (Rosenzweig and Tubiello 2007). Investigations of the response diversity by cultivars of a broader geographical origin and natural populations, in addition to locally adapted populations, such as those reported for meadow fescue (Kanapeckas et al. 2005), would be very valuable in breeding to build the adaptive capacity of forage crops. Instead of increasing only the genetic diversity of a cultivar pool, the identification of sources of response diversity to climate change ensures diversity that is more effective for adaptation of forage crops to climate change (Chapin et al. 1997; Elmqvist et al. 2003; Kahiluoto et al. 2014). Even if genetic diversity may not enhance response diversity per se (Mäkinen et al. 2015), Festulolium cultivars (synthetic *F. arundinacea* × *L. multiflorum* hybrids) exemplify the significance of a broad genetic basis for building adaptive capacity within crops (Reed and Frankham 2003; Ehlers et al. 2008). However, this type of synthetic species hybridization is most likely impractical for many other grass species. It was observed that cultivars of foreign origin (such as the timothy cultivars Lischa and Moverdi and Suez red clover) tended to respond differently when compared with the cultivars of Finnish origin. However, foreign origin inclusion was not sufficient to achieve diversity in response, as indicated by the example of the heat-resistant meadow Finnish fescue cultivar Valtteri. Our forage crop-specific results are applicable to northern European conditions, for example, by cultivating cultivars that respond differently in different fields of one farm or of one region (Himanen et al. 2013; Mäkinen et al. 2015) or by using mixtures of cultivars within the same field (Kiær et al. 2009; Tooker and Frank 2012). Further investigations that include the sensitivity of the digestibility of the forage yield to variability in critical weather components are also essential (Thorvaldsson et al. 2007; Bertrand et al. 2008).

### Global relevance

Under the intensified variability, extremes (Coumou and Rahmstorf 2012) and uncertainty (Rötter et al. 2013) of global climate change, strategies for building capacity to adapt to various plausible changes are required. Such capacity-oriented strategies complement the globally prevalent strategies to ‘predict-and-adapt’ to long-term average changes in climate that are difficult to predict (Dessai et al. 2007) as highlighted by our results. The proposed approach to assess and increase the response diversity represents a strategy to safeguard adaptation of crops without precise climate predictions, acknowledging uncertainty. Such adaptation strategies are important to apply more generally to food and feed crops that are cornerstones of global and national food security (Trnka et al. 2014). Through similar assessments as exemplified here, potential gaps in the adaptive capacity can be recognized and bridged. This is particularly important given the inherent vulnerability of the global food supply that has become relatively species-poor during the past 50 years (Khourya et al. 2014) and also nationally homogeneous worldwide.

Currently, the adaptive capacity of commercial crops is largely determined by private plant breeding companies in most regions around the world. Thus, because of the highly competitive market with a high cost pressure, the size of the cultivar market may determine the breeding goals. Consequently, some traits such as sensitivity to a particularly important weather variable might go unnoticed, which could contribute to a gap in the adaptive capacity of the cultivar pool. Specific tools for increasing the response diversity within species through breeding and diversification of cultivated crops will require further studies and the involvement of the relevant stakeholders, including both breeders and farmers (Paavola et al. 2016). Public actors, such as national emergency supply agencies or relevant ministries, should be included in setting the cultivation and breeding goals and financing breeding for the adaptive capacity in crops to provide stable food and fodder, because longer term goals may not be privately financed in a competitive market.

Regarding the presumably more frequent and intensive extreme conditions in the future (Christidis et al. 2014) anywhere around the world, an option to apply the assessment illustrated here would be to use within each weather variable a wider moderate category (such as 50 %) in the analysis, while here, each weather category (low, moderate and high) included a third of the observations. Such a modified assessment would give more weight to the capacity to adapt to extreme conditions in the response diversity assessment.

## Conclusions

Cultivars should be developed for adaptation to the variability of and extremes in weather and for multiple scenarios for mid- and long-term perspectives for global climate change. In the present study, empirical assessments of the sensitivity of yield of a modern set of forage cultivars to a broad range of critical weather conditions revealed the adaptive capacity of the cultivar pool and also its weak points; thus, such empirical assessments can be a practical tool for breeding for an uncertain future. Whereas the low adaptive capacity of timothy and meadow fescue indicate the development of more diverse breeding material is warranted, the set of festulolium cultivars was a good example of enhanced adaptive capacity to climate change through diversification of the cultivar germplasm base, even if synthetic species hybridization such as taken place for festulolium is most likely impractical for many other grass species. Furthermore, the inclusion of foreign origin in a cultivar pool was not necessary to ensure added value for response diversity. Similar analyses as exemplified here are useful regarding the entire set of food and feed crops, to secure the capacity to adapt to the variability and changes driven by global climate change. Diversity in responses to weather as one example of using within-species variation to increase the capacity to adapt to global climate change (Carpenter et al. 2015) can contribute to an increase in food security. Therefore, practical tools based on similar empirical assessments are required for breeders, farmers and public actors, as well as for practitioners on many other fields around the globe.

## Compliance with ethical standards

Online Resource 1 (DOCX 169 kb)
