# Supplementary material for: Gaps in the capacity of modern forage crops to adapt to the changing climate in northern Europe
Source: Mitig Adapt Strateg Glob Chang. 2016 Dec 7;23(1):81–100. doi: 10.1007/s11027-016-9729-5 (PMC6054012; doi:10.1007/s11027-016-9729-5)
Supplement: Online Resource 1 — (DOCX 169 kb) [file 11027_2016_9729_MOESM1_ESM.docx]

**APPENDIX**

**Gaps in the capacity of modern forage crops to adapt to the changing climate in northern Europe**

Online resources 1. The set of tested modern cultivars of timothy, meadow fescue, tall fescue and red clover. Names of the cultivars and the corresponding holders.

| Timothy | Holder | Meadow fescue | Holder | Festulolium | Holder | Tall fescue | Holder | Red clover | Holder |
| --- | --- | --- | --- | --- | --- | --- | --- | --- | --- |
| BOR 2002 | Bor | BOR 20203 | Bor | FELINA (6x CZ) | DLF | KAROLIINA | Bor | PERTTULI | Jouni Kumpulainen |
| BOR 2003 | Bor | BOR 836 | Bor | FOJTAN (6x CZ) | DLF | KORA | DLF | SUEZ | DLF |
| BOR 2005 | Bor | FP 6 | CEBECO | HYKOR (6x CZ) | DLF | SWAJ | SW | SW ARES | SW |
| BOR 94391 | Bor | INKERI | Bor |  |  |  |  | SW TORUN | SW |
| DP 70-9802 | DLF | KLAARA | Bor |  |  |  |  | SW YNGVE | SW |
| LIDAR | DLF | LIFARA | EG/SSD |  |  |  |  |  |  |
| LINUS | EG | REVANSCH | SW |  |  |  |  |  |  |
| LISCHA | EG/SSD | SW MINTO | SW |  |  |  |  |  |  |
| MOVERDI | DLF | VALTTERI | Bor |  |  |  |  |  |  |
| NIILO | Bor |  |  |  |  |  |  |  |  |
| NUUTTI | Bor |  |  |  |  |  |  |  |  |
| RAGNAR | SW |  |  |  |  |  |  |  |  |
| RAKEL | SW |  |  |  |  |  |  |  |  |
| RHONIA | Bor |  |  |  |  |  |  |  |  |
| RIGEL | Bor |  |  |  |  |  |  |  |  |
| RUBINA | Bor |  | Bor, Boreal Plant Breeding Ltd, Finland; DLF, DLF–Trifolium A/S, Denmark; CEBECO; NL, Cebeco Zalden B.V.; GER | | | | | |  |
| SWITCH | SW |  | EG, EG Euro Grass Breeding GmbH & Co.; SSD, Scandinavian Seed AB.,  Sverige; SW, Lantmännen SW Seed AB, Sweden. | | | | | | |
| TENHO | Bor |  |  | | |  |  |  |  |
| TRYGGVE | SW |  |  |  |  |  |  |  |  |

Abbreviations

FH Length of fall hardening period, days

FH-COLD Accumulation of cold temperatures during FH <5 °C, degree-days

FH-RAIN Mean daily rainfall during FH, mm

W-THAW Mean daily accumulation of temperature >0 °C during WP, degree-days

W-STRESS Accumulation of cold stress days with temperature < -15 °C, days

GP-DD5 Temperature sum >5 °C, degree-days

GP-TEMP Mean daily temperature sum accumulation rate, degree-days

GP-TEMP28 Number of days with maximum temperature of 28°C, days

GP-TEMP25_1 Number of days with maximum temperature of 25 °C from GP start to 1st cut, days

GP-TEMP25_2 Number of days with maximum temperature of 25°C from 1st cut to 2nd cut, days

GP-TEMP_2_7 Accumulation of temperature sum 7 days after 1st cut, degree-days

GP-RAIN_1 Accumulation of precipitation from GP start to 1st cut, mm

GP-RAIN_2 Accumulation of precipitation from 1st cut to 2nd cut, mm

GP-RAIN_14 Accumulation of precipitation 14 days after 1st cut, mm

Online resources 2. The effects of the tested agro-climatic variables to the yields of timothy cultivars.

| Agro-climatic variable | Cultivar | Category | Yield % of average | Mean |
| --- | --- | --- | --- | --- |
| GP-DD5 | BOR 2002 | 1 | 95 | 8 848 |
|  |  | 2 | 110 | 10 199 |
|  |  | 3 | 105 | 9 759 |
|  | BOR 2003 | 1 | 96 | 8 871 |
|  |  | 2 | 110 | 10 204 |
|  |  | 3 | 107 | 9 899 |
|  | BOR 2005 | 1 | 95 | 8 774 |
|  |  | 2 | 112 | 10 376 |
|  |  | 3 | 106 | 9 853 |
|  | BOR 94391 | 1 | 95 | 8 778 |
|  |  | 2 | 102 | 9 485 |
|  |  | 3 | 101 | 9 378 |
|  | DP 70-9802 | 1 | 88 | 8 143 |
|  |  | 2 | 108 | 10 052 |
|  |  | 3 | 104 | 9 677 |
|  | LIDAR | 1 | 97 | 8 989 |
|  |  | 2 | 107 | 9 972 |
|  |  | 3 | 107 | 9 920 |
|  | LINUS | 1 | 86 | 8 025 |
|  |  | 2 | 94 | 8 741 |
|  |  | 3 | 98 | 9 077 |
|  | LISCHA | 1 | 82 | 7 633 |
|  |  | 2 | 96 | 8 941 |
|  |  | 3 | 102 | 9 448 |
|  | MOVERDI | 1 | 70 | 6 501 |
|  |  | 2 | 93 | 8 662 |
|  |  | 3 | 95 | 8 859 |
|  | NIILO | 1 | 90 | 8 402 |
|  |  | 2 | 103 | 9 516 |
|  |  | 3 | 102 | 9 491 |
|  | NUUTTI | 1 | 93 | 8 630 |
|  |  | 2 | 110 | 10 217 |
|  |  | 3 | 106 | 9 803 |
|  | RAGNAR | 1 | 83 | 7 662 |
|  |  | 2 | 93 | 8 680 |
|  |  | 3 | 93 | 8 641 |
|  | RAKEL | 1 | 100 | 9 319 |
|  |  | 2 | 115 | 10 716 |
|  |  | 3 | 110 | 10 254 |
|  | RHONIA | 1 | 99 | 9 222 |
|  |  | 2 | 105 | 9 716 |
|  |  | 3 | 109 | 10 134 |
|  | RIGEL | 1 | 94 | 8 702 |
|  |  | 2 | 106 | 9 829 |
|  |  | 3 | 107 | 9 951 |
|  | RUBINA | 1 | 100 | 9 301 |
|  |  | 2 | 110 | 10 191 |
|  |  | 3 | 109 | 10 143 |
|  | SWITCH | 1 | 95 | 8 777 |
|  |  | 2 | 102 | 9 430 |
|  |  | 3 | 107 | 9 919 |
|  | TENHO | 1 | 91 | 8 439 |
|  |  | 2 | 104 | 9 646 |
|  |  | 3 | 103 | 9 600 |
|  | TRYGGVE | 1 | 90 | 8 379 |
|  |  | 2 | 108 | 10 064 |
|  |  | 3 | 101 | 9 332 |
| GP-TEMP_2_7 | BOR 2002 | 1 | 107 | 9 701 |
|  |  | 2 | 99 | 8 987 |
|  |  | 3 | 100 | 9 096 |
|  | BOR 2003 | 1 | 107 | 9 676 |
|  |  | 2 | 101 | 9 168 |
|  |  | 3 | 102 | 9 228 |
|  | BOR 2005 | 1 | 104 | 9 477 |
|  |  | 2 | 99 | 9 011 |
|  |  | 3 | 104 | 9 434 |
|  | BOR 94391 | 1 | 113 | 10 253 |
|  |  | 2 | 97 | 8 846 |
|  |  | 3 | 103 | 9 385 |
|  | DP 70-9802 | 1 | 102 | 9 279 |
|  |  | 2 | 94 | 8 572 |
|  |  | 3 | 97 | 8 816 |
|  | LIDAR | 1 | 103 | 9 387 |
|  |  | 2 | 105 | 9 545 |
|  |  | 3 | 112 | 10 215 |
|  | LINUS | 1 | 90 | 8 190 |
|  |  | 2 | 92 | 8 326 |
|  |  | 3 | 93 | 8 424 |
|  | LISCHA | 1 | 112 | 10 189 |
|  |  | 2 | 93 | 8 450 |
|  |  | 3 | 78 | 7 048 |
|  | MOVERDI | 1 | 81 | 7 350 |
|  |  | 2 | 88 | 8 032 |
|  |  | 3 | 88 | 7 996 |
|  | NIILO | 1 | 103 | 9 336 |
|  |  | 2 | 95 | 8 628 |
|  |  | 3 | 99 | 9 012 |
|  | NUUTTI | 1 | 103 | 9 402 |
|  |  | 2 | 101 | 9 221 |
|  |  | 3 | 99 | 8 959 |
|  | RAGNAR | 1 | 92 | 8 392 |
|  |  | 2 | 83 | 7 570 |
|  |  | 3 | 96 | 8 715 |
|  | RAKEL | 1 | 111 | 10 080 |
|  |  | 2 | 106 | 9 608 |
|  | RHONIA | 1 | 106 | 9 638 |
|  |  | 2 | 103 | 9 382 |
|  |  | 3 | 103 | 9 383 |
|  | RIGEL | 1 | 103 | 9 401 |
|  |  | 2 | 100 | 9 126 |
|  |  | 3 | 102 | 9 276 |
|  | RUBINA | 1 | 107 | 9 733 |
|  |  | 2 | 106 | 9 662 |
|  |  | 3 | 109 | 9 928 |
|  | SWITCH | 1 | 101 | 9 217 |
|  |  | 2 | 101 | 9 201 |
|  |  | 3 | 107 | 9 750 |
|  | TENHO | 1 | 100 | 9 089 |
|  |  | 2 | 99 | 9 007 |
|  |  | 3 | 98 | 8 935 |
|  | TRYGGVE | 1 | 100 | 9 122 |
|  |  | 2 | 98 | 8 869 |
|  |  | 3 | 100 | 9 043 |
| GP-TEMP | BOR 2002 | 1 | 102 | 9 365 |
|  |  | 2 | 98 | 8 998 |
|  |  | 3 | 107 | 9 803 |
|  | BOR 2003 | 1 | 102 | 9 370 |
|  |  | 2 | 103 | 9 430 |
|  |  | 3 | 107 | 9 797 |
|  | BOR 2005 | 1 | 97 | 8 890 |
|  |  | 2 | 101 | 9 206 |
|  |  | 3 | 110 | 10 090 |
|  | BOR 94391 | 1 | 101 | 9 205 |
|  |  | 2 | 99 | 9 019 |
|  |  | 3 | 103 | 9 465 |
|  | DP 70-9802 | 1 | 94 | 8 628 |
|  |  | 2 | 98 | 9 007 |
|  |  | 3 | 105 | 9 602 |
|  | LIDAR | 1 | 99 | 9 076 |
|  |  | 2 | 105 | 9 636 |
|  |  | 3 | 108 | 9 903 |
|  | LINUS | 1 | 94 | 8 591 |
|  |  | 2 | 88 | 8 014 |
|  |  | 3 | 98 | 8 968 |
|  | LISCHA | 1 | 84 | 7 722 |
|  |  | 2 | 94 | 8 572 |
|  |  | 3 | 98 | 8 986 |
|  | MOVERDI | 1 | 74 | 6 736 |
|  |  | 2 | 90 | 8 279 |
|  |  | 3 | 92 | 8 437 |
|  | NIILO | 1 | 98 | 8 935 |
|  |  | 2 | 98 | 8 950 |
|  |  | 3 | 101 | 9 240 |
|  | NUUTTI | 1 | 101 | 9 275 |
|  |  | 2 | 101 | 9 256 |
|  |  | 3 | 105 | 9 625 |
|  | RAGNAR | 1 | 80 | 7 354 |
|  |  | 2 | 90 | 8 245 |
|  |  | 3 | 99 | 9 037 |
|  | RAKEL | 1 | 106 | 9 738 |
|  |  | 2 | 109 | 9 989 |
|  |  | 3 | 113 | 10 317 |
|  | RHONIA | 1 | 99 | 9 107 |
|  |  | 2 | 108 | 9 856 |
|  |  | 3 | 109 | 9 982 |
|  | RIGEL | 1 | 98 | 8 985 |
|  |  | 2 | 103 | 9 404 |
|  |  | 3 | 106 | 9 694 |
|  | RUBINA | 1 | 103 | 9 474 |
|  |  | 2 | 108 | 9 887 |
|  |  | 3 | 110 | 10 061 |
|  | SWITCH | 1 | 95 | 8 659 |
|  |  | 2 | 107 | 9 771 |
|  |  | 3 | 106 | 9 669 |
|  | TENHO | 1 | 95 | 8 724 |
|  |  | 2 | 99 | 9 094 |
|  |  | 3 | 104 | 9 485 |
|  | TRYGGVE | 1 | 96 | 8 758 |
|  |  | 2 | 96 | 8 806 |
|  |  | 3 | 105 | 9 633 |
| GP-RAIN_14 | BOR 2002 | 1 | 103 | 9 341 |
|  |  | 2 | 104 | 9 506 |
|  |  | 3 | 101 | 9 198 |
|  | BOR 2003 | 1 | 103 | 9 395 |
|  |  | 2 | 105 | 9 532 |
|  |  | 3 | 102 | 9 331 |
|  | BOR 2005 | 1 | 102 | 9 273 |
|  |  | 2 | 103 | 9 389 |
|  |  | 3 | 103 | 9 342 |
|  | BOR 94391 | 1 | 100 | 9 126 |
|  |  | 2 | 111 | 10 132 |
|  |  | 3 | 98 | 8 965 |
|  | DP 70-9802 | 1 | 102 | 9 298 |
|  |  | 2 | 100 | 9 136 |
|  |  | 3 | 95 | 8 644 |
|  | LIDAR | 1 | 106 | 9 618 |
|  |  | 2 | 104 | 9 487 |
|  |  | 3 | 101 | 9 234 |
|  | LINUS | 1 | 93 | 8 496 |
|  |  | 2 | 91 | 8 304 |
|  |  | 3 | 91 | 8 273 |
|  | LISCHA | 1 | 91 | 8 318 |
|  |  | 2 | 111 | 10 068 |
|  |  | 3 | 90 | 8 197 |
|  | MOVERDI | 1 | 88 | 8 001 |
|  |  | 2 | 90 | 8 225 |
|  |  | 3 | 71 | 6 485 |
|  | NIILO | 1 | 98 | 8 946 |
|  |  | 2 | 97 | 8 841 |
|  |  | 3 | 98 | 8 917 |
|  | NUUTTI | 1 | 99 | 9 017 |
|  |  | 2 | 104 | 9 492 |
|  |  | 3 | 101 | 9 205 |
|  | RAGNAR | 1 | 92 | 8 384 |
|  |  | 2 | 88 | 8 006 |
|  |  | 3 | 93 | 8 474 |
|  | RAKEL | 1 | 107 | 9 762 |
|  |  | 2 | 109 | 9 935 |
|  |  | 3 | 110 | 9 972 |
|  | RHONIA | 1 | 106 | 9 632 |
|  |  | 2 | 101 | 9 195 |
|  |  | 3 | 107 | 9 756 |
|  | RIGEL | 1 | 104 | 9 499 |
|  |  | 2 | 101 | 9 172 |
|  |  | 3 | 103 | 9 345 |
|  | RUBINA | 1 | 107 | 9 778 |
|  |  | 2 | 104 | 9 468 |
|  |  | 3 | 109 | 9 886 |
|  | SWITCH | 1 | 105 | 9 559 |
|  |  | 2 | 98 | 8 907 |
|  |  | 3 | 102 | 9 245 |
|  | TENHO | 1 | 101 | 9 196 |
|  |  | 2 | 98 | 8 917 |
|  |  | 3 | 99 | 8 977 |
|  | TRYGGVE | 1 | 101 | 9 231 |
|  |  | 2 | 99 | 9 027 |
|  |  | 3 | 99 | 8 976 |
| GP-RAIN_1 | BOR 2002 | 1 | 102 | 9 286 |
|  |  | 2 | 105 | 9 474 |
|  |  | 3 | 103 | 9 334 |
|  | BOR 2003 | 1 | 105 | 9 545 |
|  |  | 2 | 104 | 9 427 |
|  |  | 3 | 102 | 9 242 |
|  | BOR 2005 | 1 | 105 | 9 478 |
|  |  | 2 | 102 | 9 267 |
|  |  | 3 | 102 | 9 239 |
|  | BOR 94391 | 1 | 96 | 8 733 |
|  |  | 2 | 106 | 9 628 |
|  |  | 3 | 97 | 8 785 |
|  | DP 70-9802 | 1 | 103 | 9 322 |
|  |  | 2 | 95 | 8 619 |
|  |  | 3 | 102 | 9 248 |
|  | LIDAR | 1 | 105 | 9 508 |
|  |  | 2 | 102 | 9 270 |
|  |  | 3 | 104 | 9 457 |
|  | LINUS | 1 | 89 | 8 110 |
|  |  | 2 | 92 | 8 348 |
|  |  | 3 | 95 | 8 625 |
|  | LISCHA | 1 | 99 | 8 948 |
|  |  | 2 | 92 | 8 305 |
|  |  | 3 | 88 | 7 981 |
|  | MOVERDI | 1 | 86 | 7 827 |
|  |  | 2 | 78 | 7 112 |
|  |  | 3 | 91 | 8 278 |
|  | NIILO | 1 | 98 | 8 861 |
|  |  | 2 | 101 | 9 124 |
|  |  | 3 | 96 | 8 710 |
|  | NUUTTI | 1 | 102 | 9 282 |
|  |  | 2 | 103 | 9 301 |
|  |  | 3 | 100 | 9 081 |
|  | RAGNAR | 1 | 96 | 8 659 |
|  |  | 2 | 91 | 8 247 |
|  |  | 3 | 88 | 7 992 |
|  | RAKEL | 1 | 107 | 9 662 |
|  |  | 2 | 110 | 10 010 |
|  |  | 3 | 110 | 9 961 |
|  | RHONIA | 1 | 103 | 9 296 |
|  |  | 2 | 108 | 9 804 |
|  |  | 3 | 105 | 9 525 |
|  | RIGEL | 1 | 101 | 9 140 |
|  |  | 2 | 106 | 9 605 |
|  |  | 3 | 101 | 9 131 |
|  | RUBINA | 1 | 105 | 9 530 |
|  |  | 2 | 110 | 9 947 |
|  |  | 3 | 106 | 9 627 |
|  | SWITCH | 1 | 102 | 9 232 |
|  |  | 2 | 102 | 9 253 |
|  |  | 3 | 100 | 9 074 |
|  | TENHO | 1 | 98 | 8 858 |
|  |  | 2 | 100 | 9 098 |
|  |  | 3 | 100 | 9 089 |
|  | TRYGGVE | 1 | 99 | 8 961 |
|  |  | 2 | 100 | 9 085 |
|  |  | 3 | 100 | 9 021 |
| GP-RAIN_2 | BOR 2002 | 1 | 98 | 8 876 |
|  |  | 2 | 109 | 9 876 |
|  |  | 3 | 99 | 8 948 |
|  | BOR 2003 | 1 | 98 | 8 913 |
|  |  | 2 | 110 | 9 929 |
|  |  | 3 | 101 | 9 161 |
|  | BOR 2005 | 1 | 100 | 9 074 |
|  |  | 2 | 106 | 9 625 |
|  |  | 3 | 102 | 9 200 |
|  | BOR 94391 | 1 | 96 | 8 718 |
|  |  | 2 | 100 | 9 080 |
|  |  | 3 | 102 | 9 215 |
|  | DP 70-9802 | 1 | 97 | 8 756 |
|  |  | 2 | 105 | 9 477 |
|  |  | 3 | 97 | 8 794 |
|  | LIDAR | 1 | 102 | 9 236 |
|  |  | 2 | 107 | 9 683 |
|  |  | 3 | 103 | 9 309 |
|  | LINUS | 1 | 93 | 8 390 |
|  |  | 2 | 94 | 8 516 |
|  |  | 3 | 93 | 8 392 |
|  | LISCHA | 1 | 98 | 8 905 |
|  |  | 2 | 90 | 8 160 |
|  |  | 3 | 91 | 8 233 |
|  | MOVERDI | 1 | 94 | 8 501 |
|  |  | 2 | 89 | 8 108 |
|  |  | 3 | 76 | 6 892 |
|  | NIILO | 1 | 96 | 8 697 |
|  |  | 2 | 100 | 9 045 |
|  |  | 3 | 100 | 9 054 |
|  | NUUTTI | 1 | 98 | 8 851 |
|  |  | 2 | 106 | 9 586 |
|  |  | 3 | 102 | 9 229 |
|  | RAGNAR | 1 | 87 | 7 871 |
|  |  | 2 | 93 | 8 469 |
|  |  | 3 | 93 | 8 447 |
|  | RAKEL | 1 | 107 | 9 733 |
|  |  | 2 | 112 | 10 124 |
|  |  | 3 | 109 | 9 866 |
|  | RHONIA | 1 | 102 | 9 256 |
|  |  | 2 | 105 | 9 528 |
|  |  | 3 | 110 | 9 945 |
|  | RIGEL | 1 | 98 | 8 845 |
|  |  | 2 | 107 | 9 658 |
|  |  | 3 | 103 | 9 351 |
|  | RUBINA | 1 | 102 | 9 206 |
|  |  | 2 | 110 | 9 926 |
|  |  | 3 | 111 | 10 082 |
|  | SWITCH | 1 | 102 | 9 261 |
|  |  | 2 | 102 | 9 216 |
|  |  | 3 | 101 | 9 173 |
|  | TENHO | 1 | 95 | 8 575 |
|  |  | 2 | 103 | 9 343 |
|  |  | 3 | 100 | 9 087 |
|  | TRYGGVE | 1 | 98 | 8 867 |
|  |  | 2 | 102 | 9 280 |
|  |  | 3 | 99 | 9 010 |
| GP-TEMP25_1 | BOR 2002 | 1 | 112 | 10 040 |
|  |  | 2 | 103 | 9 218 |
|  |  | 3 | 95 | 8 537 |
|  | BOR 2003 | 1 | 111 | 9 925 |
|  |  | 2 | 104 | 9 358 |
|  |  | 3 | 97 | 8 694 |
|  | BOR 2005 | 1 | 109 | 9 765 |
|  |  | 2 | 101 | 9 049 |
|  |  | 3 | 101 | 9 009 |
|  | BOR 94391 | 1 | 108 | 9 700 |
|  |  | 2 | 93 | 8 309 |
|  |  | 3 | 96 | 8 606 |
|  | DP 70-9802 | 1 | 102 | 9 104 |
|  |  | 2 | 102 | 9 154 |
|  |  | 3 | 94 | 8 450 |
|  | LIDAR | 1 | 109 | 9 740 |
|  |  | 2 | 106 | 9 472 |
|  |  | 3 | 101 | 9 023 |
|  | LINUS | 1 | 100 | 8 920 |
|  |  | 2 | 92 | 8 276 |
|  |  | 3 | 86 | 7 664 |
|  | LISCHA | 1 | 95 | 8 467 |
|  |  | 2 | 97 | 8 727 |
|  |  | 3 | 99 | 8 823 |
|  | MOVERDI | 1 | 83 | 7 389 |
|  |  | 2 | 89 | 7 954 |
|  |  | 3 | 83 | 7 412 |
|  | NIILO | 1 | 106 | 9 509 |
|  |  | 2 | 96 | 8 618 |
|  |  | 3 | 92 | 8 218 |
|  | NUUTTI | 1 | 108 | 9 689 |
|  |  | 2 | 102 | 9 116 |
|  |  | 3 | 97 | 8 645 |
|  | RAGNAR | 1 | 99 | 8 848 |
|  |  | 2 | 85 | 7 634 |
|  |  | 3 | 86 | 7 712 |
|  | RAKEL | 1 | 118 | 10 596 |
|  |  | 3 | 102 | 9 164 |
|  | RHONIA | 1 | 113 | 10 155 |
|  |  | 2 | 99 | 8 887 |
|  |  | 3 | 101 | 9 059 |
|  | RIGEL | 1 | 108 | 9 669 |
|  |  | 2 | 103 | 9 222 |
|  |  | 3 | 97 | 8 688 |
|  | RUBINA | 1 | 115 | 10 293 |
|  |  | 2 | 106 | 9 484 |
|  |  | 3 | 100 | 8 968 |
|  | SWITCH | 1 | 108 | 9 667 |
|  |  | 2 | 99 | 8 847 |
|  |  | 3 | 98 | 8 768 |
|  | TENHO | 1 | 106 | 9 508 |
|  |  | 2 | 99 | 8 829 |
|  |  | 3 | 95 | 8 479 |
|  | TRYGGVE | 1 | 105 | 9 432 |
|  |  | 2 | 96 | 8 569 |
|  |  | 3 | 95 | 8 498 |
| GP-TEMP25_2 | BOR 2002 | 1 | 101 | 9 131 |
|  |  | 2 | 104 | 9 365 |
|  |  | 3 | 104 | 9 390 |
|  | BOR 2003 | 1 | 101 | 9 081 |
|  |  | 2 | 107 | 9 661 |
|  |  | 3 | 105 | 9 442 |
|  | BOR 2005 | 1 | 95 | 8 587 |
|  |  | 2 | 106 | 9 571 |
|  |  | 3 | 107 | 9 636 |
|  | BOR 94391 | 1 | 98 | 8 843 |
|  |  | 2 | 100 | 8 987 |
|  |  | 3 | 103 | 9 306 |
|  | DP 70-9802 | 1 | 93 | 8 411 |
|  |  | 2 | 101 | 9 129 |
|  |  | 3 | 104 | 9 374 |
|  | LIDAR | 1 | 98 | 8 816 |
|  |  | 2 | 110 | 9 953 |
|  |  | 3 | 106 | 9 601 |
|  | LINUS | 1 | 92 | 8 267 |
|  |  | 2 | 92 | 8 286 |
|  |  | 3 | 96 | 8 619 |
|  | LISCHA | 1 | 79 | 7 088 |
|  |  | 2 | 97 | 8 782 |
|  |  | 3 | 95 | 8 569 |
|  | MOVERDI | 1 | 76 | 6 824 |
|  |  | 2 | 88 | 7 898 |
|  |  | 3 | 92 | 8 311 |
|  | NIILO | 1 | 97 | 8 726 |
|  |  | 2 | 98 | 8 842 |
|  |  | 3 | 101 | 9 074 |
|  | NUUTTI | 1 | 100 | 9 002 |
|  |  | 2 | 103 | 9 248 |
|  |  | 3 | 103 | 9 327 |
|  | RAGNAR | 1 | 77 | 6 930 |
|  |  | 2 | 98 | 8 881 |
|  |  | 3 | 94 | 8 484 |
|  | RAKEL | 1 | 111 | 10 046 |
|  |  | 2 | 111 | 10 041 |
|  |  | 3 | 111 | 10 032 |
|  | RHONIA | 1 | 99 | 8 915 |
|  |  | 2 | 109 | 9 828 |
|  |  | 3 | 108 | 9 759 |
|  | RIGEL | 1 | 98 | 8 819 |
|  |  | 2 | 104 | 9 407 |
|  |  | 3 | 105 | 9 500 |
|  | RUBINA | 1 | 103 | 9 281 |
|  |  | 2 | 109 | 9 822 |
|  |  | 3 | 109 | 9 860 |
|  | SWITCH | 1 | 94 | 8 501 |
|  |  | 2 | 109 | 9 789 |
|  |  | 3 | 104 | 9 353 |
|  | TENHO | 1 | 95 | 8 561 |
|  |  | 2 | 101 | 9 083 |
|  |  | 3 | 103 | 9 245 |
|  | TRYGGVE | 1 | 95 | 8 558 |
|  |  | 2 | 98 | 8 825 |
|  |  | 3 | 104 | 9 349 |
| GP-TEMP28 | BOR 2002 | 1 | 100 | 9 367 |
|  |  | 2 | 100 | 9 330 |
|  |  | 3 | 108 | 10 133 |
|  | BOR 2003 | 1 | 101 | 9 410 |
|  |  | 2 | 100 | 9 323 |
|  |  | 3 | 112 | 10 433 |
|  | BOR 2005 | 1 | 98 | 9 161 |
|  |  | 2 | 100 | 9 368 |
|  |  | 3 | 110 | 10 297 |
|  | BOR 94391 | 1 | 98 | 9 191 |
|  |  | 2 | 94 | 8 758 |
|  |  | 3 | 109 | 10 164 |
|  | DP 70-9802 | 1 | 92 | 8 592 |
|  |  | 2 | 97 | 9 041 |
|  |  | 3 | 111 | 10 404 |
|  | LIDAR | 1 | 100 | 9 329 |
|  |  | 2 | 100 | 9 338 |
|  |  | 3 | 112 | 10 508 |
|  | LINUS | 1 | 90 | 8 383 |
|  |  | 2 | 88 | 8 179 |
|  |  | 3 | 103 | 9 651 |
|  | LISCHA | 1 | 88 | 8 244 |
|  |  | 2 | 90 | 8 437 |
|  |  | 3 | 99 | 9 215 |
|  | MOVERDI | 1 | 73 | 6 818 |
|  |  | 2 | 84 | 7 867 |
|  |  | 3 | 102 | 9 575 |
|  | NIILO | 1 | 96 | 8 985 |
|  |  | 2 | 93 | 8 713 |
|  |  | 3 | 105 | 9 854 |
|  | NUUTTI | 1 | 99 | 9 218 |
|  |  | 2 | 99 | 9 243 |
|  |  | 3 | 109 | 10 165 |
|  | RAGNAR | 1 | 86 | 8 083 |
|  |  | 2 | 87 | 8 167 |
|  |  | 3 | 97 | 9 088 |
|  | RAKEL | 1 | 107 | 10 015 |
|  |  | 2 | 109 | 10 190 |
|  |  | 3 | 114 | 10 616 |
|  | RHONIA | 1 | 101 | 9 419 |
|  |  | 2 | 100 | 9 355 |
|  |  | 3 | 113 | 10 544 |
|  | RIGEL | 1 | 98 | 9 203 |
|  |  | 2 | 100 | 9 338 |
|  |  | 3 | 108 | 10 100 |
|  | RUBINA | 1 | 104 | 9 702 |
|  |  | 2 | 104 | 9 717 |
|  |  | 3 | 112 | 10 452 |
|  | SWITCH | 1 | 98 | 9 116 |
|  |  | 2 | 98 | 9 114 |
|  |  | 3 | 110 | 10 252 |
|  | TENHO | 1 | 95 | 8 908 |
|  |  | 2 | 97 | 9 042 |
|  |  | 3 | 106 | 9 880 |
|  | TRYGGVE | 1 | 94 | 8 770 |
|  |  | 2 | 98 | 9 136 |
|  |  | 3 | 106 | 9 902 |
| FH | BOR 2002 | 1 | 108 | 9 791 |
|  |  | 2 | 102 | 9 266 |
|  |  | 3 | 99 | 8 992 |
|  | BOR 2003 | 1 | 108 | 9 749 |
|  |  | 2 | 105 | 9 484 |
|  |  | 3 | 102 | 9 208 |
|  | BOR 2005 | 1 | 108 | 9 750 |
|  |  | 2 | 102 | 9 193 |
|  |  | 3 | 99 | 8 999 |
|  | BOR 94391 | 1 | 105 | 9 492 |
|  |  | 2 | 96 | 8 720 |
|  |  | 3 | 100 | 9 096 |
|  | DP 70-9802 | 1 | 103 | 9 315 |
|  |  | 2 | 100 | 9 086 |
|  |  | 3 | 95 | 8 605 |
|  | LIDAR | 1 | 106 | 9 589 |
|  |  | 2 | 104 | 9 390 |
|  |  | 3 | 101 | 9 157 |
|  | LINUS | 1 | 98 | 8 906 |
|  |  | 2 | 90 | 8 190 |
|  |  | 3 | 89 | 8 066 |
|  | LISCHA | 1 | 100 | 9 030 |
|  |  | 2 | 91 | 8 215 |
|  |  | 3 | 79 | 7 108 |
|  | MOVERDI | 1 | 88 | 7 979 |
|  |  | 2 | 93 | 8 397 |
|  |  | 3 | 79 | 7 147 |
|  | NIILO | 1 | 102 | 9 187 |
|  |  | 2 | 95 | 8 625 |
|  |  | 3 | 98 | 8 833 |
|  | NUUTTI | 1 | 107 | 9 684 |
|  |  | 2 | 105 | 9 543 |
|  |  | 3 | 99 | 8 917 |
|  | RAGNAR | 1 | 96 | 8 644 |
|  |  | 2 | 91 | 8 196 |
|  |  | 3 | 84 | 7 623 |
|  | RAKEL | 1 | 117 | 10 628 |
|  |  | 2 | 103 | 9 293 |
|  |  | 3 | 106 | 9 603 |
|  | RHONIA | 1 | 114 | 10 287 |
|  |  | 2 | 103 | 9 358 |
|  |  | 3 | 100 | 9 029 |
|  | RIGEL | 1 | 110 | 9 959 |
|  |  | 2 | 103 | 9 313 |
|  |  | 3 | 96 | 8 731 |
|  | RUBINA | 1 | 113 | 10 231 |
|  |  | 2 | 108 | 9 752 |
|  |  | 3 | 101 | 9 136 |
|  | SWITCH | 1 | 104 | 9 431 |
|  |  | 2 | 100 | 9 090 |
|  |  | 3 | 98 | 8 909 |
|  | TENHO | 1 | 105 | 9 482 |
|  |  | 2 | 100 | 9 055 |
|  |  | 3 | 95 | 8 589 |
|  | TRYGGVE | 1 | 105 | 9 529 |
|  |  | 2 | 97 | 8 740 |
|  |  | 3 | 95 | 8 586 |
| FH-COLD | BOR 2002 | 1 | 102 | 9 344 |
|  |  | 2 | 103 | 9 393 |
|  |  | 3 | 106 | 9 723 |
|  | BOR 2003 | 1 | 103 | 9 421 |
|  |  | 2 | 105 | 9 649 |
|  |  | 3 | 103 | 9 443 |
|  | BOR 2005 | 1 | 102 | 9 375 |
|  |  | 2 | 101 | 9 268 |
|  |  | 3 | 104 | 9 573 |
|  | BOR 94391 | 1 | 97 | 8 843 |
|  |  | 2 | 101 | 9 228 |
|  |  | 3 | 107 | 9 820 |
|  | DP 70-9802 | 1 | 96 | 8 821 |
|  |  | 2 | 100 | 9 183 |
|  |  | 3 | 101 | 9 226 |
|  | LIDAR | 1 | 105 | 9 638 |
|  |  | 2 | 102 | 9 355 |
|  |  | 3 | 99 | 9 058 |
|  | LINUS | 1 | 93 | 8 488 |
|  |  | 2 | 94 | 8 639 |
|  |  | 3 | 93 | 8 499 |
|  | LISCHA | 1 | 93 | 8 516 |
|  |  | 2 | 93 | 8 479 |
|  |  | 3 | 89 | 8 171 |
|  | MOVERDI | 1 | 84 | 7 677 |
|  |  | 2 | 90 | 8 257 |
|  |  | 3 | 78 | 7 149 |
|  | NIILO | 1 | 97 | 8 862 |
|  |  | 2 | 99 | 9 064 |
|  |  | 3 | 101 | 9 269 |
|  | NUUTTI | 1 | 100 | 9 174 |
|  |  | 2 | 104 | 9 562 |
|  |  | 3 | 104 | 9 525 |
|  | RAGNAR | 1 | 88 | 8 048 |
|  |  | 2 | 96 | 8 759 |
|  |  | 3 | 90 | 8 282 |
|  | RAKEL | 1 | 110 | 10 067 |
|  |  | 2 | 105 | 9 642 |
|  |  | 3 | 112 | 10 226 |
|  | RHONIA | 1 | 105 | 9 652 |
|  |  | 2 | 102 | 9 360 |
|  |  | 3 | 116 | 10 612 |
|  | RIGEL | 1 | 102 | 9 370 |
|  |  | 2 | 102 | 9 376 |
|  |  | 3 | 101 | 9 253 |
|  | RUBINA | 1 | 106 | 9 735 |
|  |  | 2 | 105 | 9 613 |
|  |  | 3 | 114 | 10 413 |
|  | SWITCH | 1 | 103 | 9 451 |
|  |  | 2 | 98 | 8 972 |
|  |  | 3 | 100 | 9 173 |
|  | TENHO | 1 | 99 | 9 101 |
|  |  | 2 | 99 | 9 087 |
|  |  | 3 | 100 | 9 140 |
|  | TRYGGVE | 1 | 99 | 9 063 |
|  |  | 2 | 99 | 9 077 |
|  |  | 3 | 100 | 9 143 |
| FH-RAIN | BOR 2002 | 1 | 108 | 10 023 |
|  |  | 2 | 100 | 9 266 |
|  |  | 3 | 100 | 9 305 |
|  | BOR 2003 | 1 | 109 | 10 093 |
|  |  | 2 | 103 | 9 558 |
|  |  | 3 | 100 | 9 287 |
|  | BOR 2005 | 1 | 106 | 9 829 |
|  |  | 2 | 103 | 9 539 |
|  |  | 3 | 100 | 9 318 |
|  | BOR 94391 | 1 | 107 | 9 983 |
|  |  | 2 | 100 | 9 320 |
|  |  | 3 | 87 | 8 079 |
|  | DP 70-9802 | 1 | 97 | 9 050 |
|  |  | 2 | 99 | 9 217 |
|  |  | 3 | 100 | 9 291 |
|  | LIDAR | 1 | 108 | 10 020 |
|  |  | 2 | 105 | 9 802 |
|  |  | 3 | 99 | 9 204 |
|  | LINUS | 1 | 98 | 9 110 |
|  |  | 2 | 93 | 8 617 |
|  |  | 3 | 87 | 8 117 |
|  | LISCHA | 1 | 96 | 8 887 |
|  |  | 2 | 95 | 8 803 |
|  |  | 3 | 91 | 8 497 |
|  | MOVERDI | 1 | 86 | 7 981 |
|  |  | 2 | 80 | 7 480 |
|  |  | 3 | 84 | 7 766 |
|  | NIILO | 1 | 103 | 9 588 |
|  |  | 2 | 101 | 9 429 |
|  |  | 3 | 87 | 8 049 |
|  | NUUTTI | 1 | 106 | 9 857 |
|  |  | 2 | 104 | 9 694 |
|  |  | 3 | 97 | 9 038 |
|  | RAGNAR | 1 | 93 | 8 673 |
|  |  | 2 | 94 | 8 735 |
|  |  | 3 | 90 | 8 388 |
|  | RAKEL | 1 | 114 | 10 622 |
|  |  | 2 | 108 | 10 000 |
|  |  | 3 | 108 | 10 044 |
|  | RHONIA | 1 | 108 | 10 027 |
|  |  | 2 | 109 | 10 097 |
|  |  | 3 | 98 | 9 137 |
|  | RIGEL | 1 | 107 | 9 897 |
|  |  | 2 | 104 | 9 692 |
|  |  | 3 | 98 | 9 114 |
|  | RUBINA | 1 | 110 | 10 203 |
|  |  | 2 | 108 | 10 015 |
|  |  | 3 | 104 | 9 622 |
|  | SWITCH | 1 | 106 | 9 817 |
|  |  | 2 | 101 | 9 396 |
|  |  | 3 | 99 | 9 174 |
|  | TENHO | 1 | 102 | 9 518 |
|  |  | 2 | 100 | 9 324 |
|  |  | 3 | 98 | 9 061 |
|  | TRYGGVE | 1 | 104 | 9 664 |
|  |  | 2 | 100 | 9 254 |
|  |  | 3 | 98 | 9 081 |
| W-STRESS | BOR 2002 | 1 | 104 | 9 609 |
|  |  | 2 | 111 | 10 226 |
|  |  | 3 | 95 | 8 789 |
|  | BOR 2003 | 1 | 107 | 9 846 |
|  |  | 2 | 111 | 10 220 |
|  |  | 3 | 93 | 8 592 |
|  | BOR 2005 | 1 | 104 | 9 608 |
|  |  | 2 | 108 | 9 961 |
|  |  | 3 | 95 | 8 770 |
|  | BOR 94391 | 1 | 102 | 9 412 |
|  |  | 2 | 106 | 9 776 |
|  |  | 3 | 93 | 8 569 |
|  | DP 70-9802 | 1 | 101 | 9 340 |
|  |  | 2 | 108 | 9 901 |
|  |  | 3 | 87 | 8 053 |
|  | LIDAR | 1 | 106 | 9 719 |
|  |  | 2 | 112 | 10 300 |
|  |  | 3 | 98 | 9 027 |
|  | LINUS | 1 | 94 | 8 649 |
|  |  | 2 | 97 | 8 967 |
|  |  | 3 | 88 | 8 140 |
|  | LISCHA | 1 | 81 | 7 416 |
|  |  | 2 | 106 | 9 736 |
|  |  | 3 | 82 | 7 533 |
|  | MOVERDI | 1 | 81 | 7 460 |
|  |  | 2 | 97 | 8 917 |
|  |  | 3 | 81 | 7 443 |
|  | NIILO | 1 | 105 | 9 661 |
|  |  | 2 | 100 | 9 207 |
|  |  | 3 | 91 | 8 417 |
|  | NUUTTI | 1 | 105 | 9 676 |
|  |  | 2 | 107 | 9 818 |
|  |  | 3 | 96 | 8 851 |
|  | RAGNAR | 1 | 95 | 8 781 |
|  |  | 2 | 94 | 8 663 |
|  |  | 3 | 82 | 7 521 |
|  | RAKEL | 1 | 113 | 10 379 |
|  |  | 2 | 117 | 10 743 |
|  |  | 3 | 100 | 9 190 |
|  | RHONIA | 1 | 107 | 9 887 |
|  |  | 2 | 108 | 9 905 |
|  |  | 3 | 98 | 9 063 |
|  | RIGEL | 1 | 106 | 9 723 |
|  |  | 2 | 107 | 9 873 |
|  |  | 3 | 94 | 8 664 |
|  | RUBINA | 1 | 110 | 10 090 |
|  |  | 2 | 114 | 10 450 |
|  |  | 3 | 97 | 8 971 |
|  | SWITCH | 1 | 103 | 9 478 |
|  |  | 2 | 108 | 9 964 |
|  |  | 3 | 95 | 8 771 |
|  | TENHO | 1 | 103 | 9 445 |
|  |  | 2 | 104 | 9 580 |
|  |  | 3 | 93 | 8 540 |
|  | TRYGGVE | 1 | 101 | 9 323 |
|  |  | 2 | 105 | 9 622 |
|  |  | 3 | 92 | 8 492 |
| W-THAW | BOR 2002 | 1 | 95 | 8 733 |
|  |  | 2 | 109 | 9 931 |
|  |  | 3 | 108 | 9 912 |
|  | BOR 2003 | 1 | 96 | 8 801 |
|  |  | 2 | 111 | 10 113 |
|  |  | 3 | 109 | 9 963 |
|  | BOR 2005 | 1 | 98 | 8 926 |
|  |  | 2 | 104 | 9 506 |
|  |  | 3 | 107 | 9 802 |
|  | BOR 94391 | 1 | 98 | 8 968 |
|  |  | 2 | 101 | 9 239 |
|  |  | 3 | 104 | 9 538 |
|  | DP 70-9802 | 1 | 90 | 8 272 |
|  |  | 2 | 83 | 7 600 |
|  |  | 3 | 106 | 9 681 |
|  | LIDAR | 1 | 101 | 9 259 |
|  |  | 2 | 106 | 9 664 |
|  |  | 3 | 107 | 9 830 |
|  | LINUS | 1 | 90 | 8 260 |
|  |  | 2 | 94 | 8 597 |
|  |  | 3 | 97 | 8 888 |
|  | LISCHA | 1 | 80 | 7 337 |
|  |  | 2 | 94 | 8 630 |
|  |  | 3 | 103 | 9 437 |
|  | MOVERDI | 1 | 76 | 6 912 |
|  |  | 2 | 72 | 6 607 |
|  |  | 3 | 92 | 8 384 |
|  | NIILO | 1 | 97 | 8 884 |
|  |  | 2 | 99 | 9 072 |
|  |  | 3 | 101 | 9 265 |
|  | NUUTTI | 1 | 98 | 8 949 |
|  |  | 2 | 104 | 9 524 |
|  |  | 3 | 106 | 9 723 |
|  | RAGNAR | 1 | 84 | 7 705 |
|  |  | 2 | 94 | 8 627 |
|  |  | 3 | 95 | 8 654 |
|  | RAKEL | 1 | 101 | 9 235 |
|  |  | 2 | 110 | 10 064 |
|  |  | 3 | 114 | 10 418 |
|  | RHONIA | 1 | 104 | 9 505 |
|  |  | 2 | 110 | 10 099 |
|  |  | 3 | 107 | 9 772 |
|  | RIGEL | 1 | 97 | 8 849 |
|  |  | 2 | 107 | 9 773 |
|  |  | 3 | 106 | 9 691 |
|  | RUBINA | 1 | 103 | 9 451 |
|  |  | 2 | 112 | 10 244 |
|  |  | 3 | 109 | 9 976 |
|  | SWITCH | 1 | 102 | 9 312 |
|  |  | 2 | 102 | 9 358 |
|  |  | 3 | 104 | 9 511 |
|  | TENHO | 1 | 92 | 8 457 |
|  |  | 2 | 106 | 9 738 |
|  |  | 3 | 104 | 9 482 |
|  | TRYGGVE | 1 | 96 | 8 748 |
|  |  | 2 | 98 | 8 975 |
|  |  | 3 | 104 | 9 513 |

Online resources 3. The effects of the tested agro-climatic variables to the yields of meadow fescue cultivars.

| Agro-climatic variable | Cultivar | Category | Yield % of average | Mean |
| --- | --- | --- | --- | --- |
| GP-DD5 | BOR 20203 | 1 | 99 | 8 677 |
|  |  | 2 | 106 | 9 302 |
|  |  | 3 | 103 | 9 034 |
|  | BOR 836 | 1 | 91 | 7 978 |
|  |  | 2 | 104 | 9 103 |
|  |  | 3 | 98 | 8 598 |
|  | FP 6 | 1 | 89 | 7 797 |
|  |  | 2 | 113 | 9 911 |
|  |  | 3 | 99 | 8 723 |
|  | INKERI | 1 | 94 | 8 276 |
|  |  | 2 | 108 | 9 487 |
|  |  | 3 | 99 | 8 716 |
|  | KLAARA | 1 | 98 | 8 587 |
|  |  | 2 | 105 | 9 221 |
|  |  | 3 | 101 | 8 854 |
|  | LIFARA | 1 | 86 | 7 570 |
|  |  | 2 | 115 | 10 081 |
|  |  | 3 | 98 | 8 626 |
|  | REVANSCH | 1 | 94 | 8 212 |
|  |  | 2 | 108 | 9 520 |
|  |  | 3 | 96 | 8 464 |
|  | SW MINTO | 1 | 88 | 7 682 |
|  |  | 2 | 104 | 9 104 |
|  |  | 3 | 102 | 8 920 |
|  | VALTTERI | 1 | 93 | 8 193 |
|  |  | 2 | 110 | 9 665 |
|  |  | 3 | 99 | 8 685 |
| GP-TEMP_2_7 | BOR 20203 | 1 | 110 | 9 724 |
|  |  | 2 | 104 | 9 211 |
|  | BOR 836 | 1 | 101 | 8 900 |
|  |  | 2 | 96 | 8 516 |
|  |  | 3 | 90 | 7 960 |
|  | FP 6 | 1 | 107 | 9 456 |
|  |  | 2 | 98 | 8 658 |
|  |  | 3 | 94 | 8 353 |
|  | INKERI | 1 | 103 | 9 140 |
|  |  | 2 | 100 | 8 876 |
|  |  | 3 | 95 | 8 371 |
|  | KLAARA | 1 | 108 | 9 590 |
|  |  | 2 | 102 | 9 001 |
|  | LIFARA | 1 | 105 | 9 307 |
|  |  | 2 | 97 | 8 588 |
|  |  | 3 | 90 | 7 952 |
|  | REVANSCH | 1 | 104 | 9 252 |
|  |  | 2 | 97 | 8 557 |
|  |  | 3 | 92 | 8 189 |
|  | SW MINTO | 1 | 107 | 9 475 |
|  |  | 2 | 96 | 8 462 |
|  |  | 3 | 96 | 8 536 |
|  | VALTTERI | 1 | 107 | 9 488 |
|  |  | 2 | 99 | 8 752 |
|  |  | 3 | 103 | 9 097 |
| GP-TEMP | BOR 20203 | 1 | 109 | 9 658 |
|  |  | 2 | 107 | 9 468 |
|  |  | 3 | 98 | 8 615 |
|  | BOR 836 | 1 | 99 | 8 732 |
|  |  | 2 | 97 | 8 546 |
|  |  | 3 | 93 | 8 230 |
|  | FP 6 | 1 | 94 | 8 277 |
|  |  | 2 | 105 | 9 252 |
|  |  | 3 | 96 | 8 488 |
|  | INKERI | 1 | 101 | 8 936 |
|  |  | 2 | 101 | 8 913 |
|  |  | 3 | 97 | 8 542 |
|  | KLAARA | 1 | 108 | 9 561 |
|  |  | 2 | 105 | 9 250 |
|  |  | 3 | 96 | 8 494 |
|  | LIFARA | 1 | 93 | 8 186 |
|  |  | 2 | 109 | 9 653 |
|  |  | 3 | 94 | 8 277 |
|  | REVANSCH | 1 | 105 | 9 260 |
|  |  | 2 | 100 | 8 822 |
|  |  | 3 | 93 | 8 256 |
|  | SW MINTO | 1 | 97 | 8 603 |
|  |  | 2 | 97 | 8 561 |
|  | VALTTERI | 1 | 96 | 8 460 |
|  |  | 2 | 110 | 9 734 |
|  |  | 3 | 101 | 8 929 |
| GP-RAIN_14 | BOR 20203 | 1 | 98 | 8 890 |
|  |  | 2 | 102 | 9 322 |
|  |  | 3 | 111 | 10 118 |
|  | BOR 836 | 1 | 93 | 8 457 |
|  |  | 2 | 93 | 8 496 |
|  |  | 3 | 101 | 9 181 |
|  | FP 6 | 1 | 95 | 8 624 |
|  |  | 2 | 95 | 8 672 |
|  |  | 3 | 107 | 9 752 |
|  | INKERI | 1 | 95 | 8 605 |
|  |  | 2 | 101 | 9 189 |
|  |  | 3 | 101 | 9 194 |
|  | KLAARA | 1 | 99 | 8 981 |
|  |  | 2 | 100 | 9 126 |
|  |  | 3 | 108 | 9 841 |
|  | LIFARA | 1 | 92 | 8 414 |
|  |  | 2 | 103 | 9 417 |
|  | REVANSCH | 1 | 99 | 9 012 |
|  |  | 2 | 97 | 8 862 |
|  |  | 3 | 99 | 9 012 |
|  | SW MINTO | 1 | 107 | 9 780 |
|  |  | 2 | 101 | 9 155 |
|  |  | 3 | 99 | 9 051 |
|  | VALTTERI | 1 | 100 | 9 112 |
|  |  | 2 | 101 | 9 163 |
|  |  | 3 | 102 | 9 292 |
| GP-RAIN_1 | BOR 20203 | 1 | 103 | 9 253 |
|  |  | 2 | 103 | 9 256 |
|  |  | 3 | 104 | 9 350 |
|  | BOR 836 | 1 | 93 | 8 314 |
|  |  | 2 | 96 | 8 628 |
|  |  | 3 | 99 | 8 902 |
|  | FP 6 | 1 | 98 | 8 806 |
|  |  | 2 | 104 | 9 346 |
|  |  | 3 | 103 | 9 203 |
|  | INKERI | 1 | 97 | 8 693 |
|  |  | 2 | 98 | 8 796 |
|  |  | 3 | 103 | 9 250 |
|  | KLAARA | 1 | 102 | 9 140 |
|  |  | 2 | 100 | 8 949 |
|  |  | 3 | 104 | 9 351 |
|  | LIFARA | 1 | 98 | 8 770 |
|  |  | 2 | 100 | 8 935 |
|  |  | 3 | 99 | 8 873 |
|  | REVANSCH | 1 | 94 | 8 466 |
|  |  | 2 | 98 | 8 750 |
|  |  | 3 | 103 | 9 223 |
|  | SW MINTO | 1 | 103 | 9 200 |
|  |  | 2 | 98 | 8 815 |
|  |  | 3 | 98 | 8 776 |
|  | VALTTERI | 1 | 98 | 8 757 |
|  |  | 2 | 102 | 9 108 |
|  |  | 3 | 104 | 9 364 |
| GP-RAIN_2 | BOR 20203 | 1 | 100 | 8 987 |
|  |  | 2 | 105 | 9 465 |
|  |  | 3 | 109 | 9 784 |
|  | BOR 836 | 1 | 98 | 8 786 |
|  |  | 2 | 91 | 8 179 |
|  |  | 3 | 99 | 8 895 |
|  | FP 6 | 1 | 105 | 9 410 |
|  |  | 2 | 97 | 8 701 |
|  |  | 3 | 104 | 9 347 |
|  | INKERI | 1 | 104 | 9 318 |
|  |  | 2 | 92 | 8 316 |
|  |  | 3 | 101 | 9 069 |
|  | KLAARA | 1 | 99 | 8 866 |
|  |  | 2 | 102 | 9 140 |
|  |  | 3 | 108 | 9 761 |
|  | LIFARA | 1 | 104 | 9 327 |
|  |  | 2 | 93 | 8 390 |
|  |  | 3 | 101 | 9 112 |
|  | REVANSCH | 1 | 98 | 8 826 |
|  |  | 2 | 97 | 8 701 |
|  |  | 3 | 97 | 8 741 |
|  | SW MINTO | 1 | 101 | 9 120 |
|  |  | 2 | 96 | 8 656 |
|  |  | 3 | 99 | 8 881 |
|  | VALTTERI | 1 | 103 | 9 278 |
|  |  | 2 | 98 | 8 820 |
|  |  | 3 | 101 | 9 042 |
| GP-TEMP25_1 | BOR 20203 | 1 | 106 | 9 551 |
|  |  | 2 | 108 | 9 705 |
|  |  | 3 | 98 | 8 835 |
|  | BOR 836 | 1 | 96 | 8 652 |
|  |  | 2 | 100 | 9 023 |
|  |  | 3 | 89 | 7 999 |
|  | FP 6 | 1 | 100 | 8 978 |
|  |  | 2 | 105 | 9 441 |
|  |  | 3 | 95 | 8 501 |
|  | INKERI | 1 | 101 | 9 055 |
|  |  | 2 | 103 | 9 248 |
|  |  | 3 | 94 | 8 436 |
|  | KLAARA | 1 | 103 | 9 266 |
|  |  | 2 | 110 | 9 865 |
|  |  | 3 | 96 | 8 620 |
|  | LIFARA | 1 | 98 | 8 808 |
|  |  | 2 | 103 | 9 267 |
|  |  | 3 | 91 | 8 181 |
|  | REVANSCH | 1 | 97 | 8 762 |
|  |  | 2 | 106 | 9 511 |
|  |  | 3 | 93 | 8 389 |
|  | SW MINTO | 1 | 93 | 8 351 |
|  |  | 2 | 98 | 8 853 |
|  | VALTTERI | 1 | 101 | 9 051 |
|  |  | 2 | 102 | 9 211 |
|  |  | 3 | 114 | 10 287 |
| GP-TEMP25_2 | BOR 20203 | 1 | 110 | 9 825 |
|  |  | 2 | 103 | 9 147 |
|  |  | 3 | 96 | 8 499 |
|  | BOR 836 | 1 | 95 | 8 437 |
|  |  | 2 | 100 | 8 901 |
|  |  | 3 | 92 | 8 225 |
|  | FP 6 | 1 | 102 | 9 081 |
|  |  | 2 | 102 | 9 081 |
|  |  | 3 | 96 | 8 497 |
|  | INKERI | 1 | 97 | 8 638 |
|  |  | 2 | 106 | 9 422 |
|  |  | 3 | 96 | 8 550 |
|  | KLAARA | 1 | 106 | 9 452 |
|  |  | 2 | 106 | 9 451 |
|  |  | 3 | 91 | 8 099 |
|  | LIFARA | 1 | 101 | 8 965 |
|  |  | 2 | 102 | 9 114 |
|  |  | 3 | 91 | 8 099 |
|  | REVANSCH | 1 | 100 | 8 926 |
|  |  | 2 | 100 | 8 875 |
|  |  | 3 | 93 | 8 280 |
|  | SW MINTO | 1 | 91 | 8 137 |
|  |  | 2 | 103 | 9 168 |
|  |  | 3 | 97 | 8 654 |
|  | VALTTERI | 1 | 101 | 8 944 |
|  |  | 2 | 123 | 10 900 |
|  |  | 3 | 100 | 8 866 |
| GP-TEMP28 | BOR 20203 | 1 | 108 | 9 374 |
|  |  | 2 | 107 | 9 266 |
|  |  | 3 | 98 | 8 450 |
|  | BOR 836 | 1 | 97 | 8 415 |
|  |  | 2 | 99 | 8 542 |
|  |  | 3 | 95 | 8 256 |
|  | FP 6 | 1 | 101 | 8 761 |
|  |  | 2 | 101 | 8 714 |
|  |  | 3 | 97 | 8 375 |
|  | INKERI | 1 | 101 | 8 730 |
|  |  | 2 | 103 | 8 892 |
|  |  | 3 | 97 | 8 436 |
|  | KLAARA | 1 | 106 | 9 205 |
|  |  | 2 | 107 | 9 246 |
|  |  | 3 | 94 | 8 179 |
|  | LIFARA | 1 | 102 | 8 836 |
|  |  | 2 | 96 | 8 302 |
|  |  | 3 | 93 | 8 084 |
|  | REVANSCH | 1 | 101 | 8 721 |
|  |  | 2 | 103 | 8 921 |
|  |  | 3 | 93 | 8 066 |
|  | SW MINTO | 1 | 95 | 8 250 |
|  |  | 2 | 95 | 8 208 |
|  |  | 3 | 99 | 8 598 |
|  | VALTTERI | 1 | 99 | 8 615 |
|  |  | 2 | 108 | 9 381 |
|  |  | 3 | 105 | 9 091 |
| FH | BOR 20203 | 1 | 112 | 9 670 |
|  |  | 2 | 109 | 9 400 |
|  |  | 3 | 103 | 8 937 |
|  | BOR 836 | 1 | 94 | 8 100 |
|  |  | 2 | 97 | 8 376 |
|  |  | 3 | 96 | 8 262 |
|  | FP 6 | 1 | 96 | 8 265 |
|  |  | 2 | 95 | 8 190 |
|  |  | 3 | 102 | 8 805 |
|  | INKERI | 1 | 95 | 8 210 |
|  |  | 2 | 100 | 8 654 |
|  |  | 3 | 104 | 8 973 |
|  | KLAARA | 1 | 109 | 9 452 |
|  |  | 2 | 100 | 8 625 |
|  |  | 3 | 103 | 8 930 |
|  | LIFARA | 1 | 94 | 8 087 |
|  |  | 2 | 96 | 8 265 |
|  |  | 3 | 100 | 8 676 |
|  | REVANSCH | 1 | 98 | 8 455 |
|  |  | 2 | 98 | 8 428 |
|  |  | 3 | 100 | 8 609 |
|  | SW MINTO | 1 | 92 | 7 980 |
|  |  | 2 | 101 | 8 763 |
|  |  | 3 | 101 | 8 725 |
|  | VALTTERI | 1 | 98 | 8 482 |
|  |  | 2 | 101 | 8 766 |
|  |  | 3 | 106 | 9 193 |
| FH-COLD | BOR 20203 | 1 | 110 | 9 564 |
|  |  | 2 | 96 | 8 324 |
|  |  | 3 | 118 | 10 267 |
|  | BOR 836 | 1 | 100 | 8 723 |
|  |  | 2 | 93 | 8 091 |
|  |  | 3 | 94 | 8 215 |
|  | FP 6 | 1 | 104 | 9 063 |
|  |  | 2 | 94 | 8 167 |
|  |  | 3 | 95 | 8 315 |
|  | INKERI | 1 | 102 | 8 907 |
|  |  | 2 | 96 | 8 364 |
|  |  | 3 | 102 | 8 890 |
|  | KLAARA | 1 | 108 | 9 378 |
|  |  | 2 | 94 | 8 208 |
|  |  | 3 | 117 | 10 225 |
|  | LIFARA | 1 | 102 | 8 888 |
|  |  | 2 | 94 | 8 178 |
|  |  | 3 | 92 | 7 995 |
|  | REVANSCH | 1 | 103 | 8 981 |
|  |  | 2 | 94 | 8 166 |
|  |  | 3 | 95 | 8 308 |
|  | SW MINTO | 1 | 104 | 9 073 |
|  |  | 2 | 96 | 8 321 |
|  |  | 3 | 96 | 8 378 |
|  | VALTTERI | 1 | 108 | 9 412 |
|  |  | 2 | 95 | 8 311 |
|  |  | 3 | 96 | 8 384 |
| FH-RAIN | BOR 20203 | 1 | 98 | 8 428 |
|  |  | 2 | 107 | 9 197 |
|  |  | 3 | 110 | 9 436 |
|  | BOR 836 | 1 | 87 | 7 466 |
|  |  | 2 | 99 | 8 446 |
|  |  | 3 | 100 | 8 589 |
|  | FP 6 | 1 | 94 | 8 073 |
|  |  | 2 | 104 | 8 930 |
|  |  | 3 | 103 | 8 835 |
|  | INKERI | 1 | 94 | 8 021 |
|  |  | 2 | 104 | 8 931 |
|  |  | 3 | 101 | 8 677 |
|  | KLAARA | 1 | 96 | 8 256 |
|  |  | 2 | 107 | 9 198 |
|  |  | 3 | 106 | 9 111 |
|  | LIFARA | 1 | 90 | 7 734 |
|  |  | 2 | 106 | 9 053 |
|  |  | 3 | 108 | 9 270 |
|  | REVANSCH | 1 | 91 | 7 798 |
|  |  | 2 | 100 | 8 600 |
|  |  | 3 | 102 | 8 701 |
|  | SW MINTO | 1 | 93 | 7 991 |
|  |  | 2 | 102 | 8 779 |
|  |  | 3 | 95 | 8 108 |
|  | VALTTERI | 1 | 92 | 7 892 |
|  |  | 2 | 105 | 8 998 |
|  |  | 3 | 104 | 8 915 |
| W-STRESS | BOR 20203 | 1 | 113 | 9 596 |
|  |  | 2 | 110 | 9 409 |
|  |  | 3 | 89 | 7 579 |
|  | BOR 836 | 1 | 102 | 8 705 |
|  |  | 2 | 104 | 8 873 |
|  |  | 3 | 86 | 7 306 |
|  | FP 6 | 1 | 102 | 8 700 |
|  |  | 2 | 108 | 9 233 |
|  |  | 3 | 86 | 7 344 |
|  | INKERI | 1 | 104 | 8 900 |
|  |  | 2 | 108 | 9 194 |
|  |  | 3 | 92 | 7 844 |
|  | KLAARA | 1 | 112 | 9 508 |
|  |  | 2 | 106 | 9 054 |
|  |  | 3 | 86 | 7 348 |
|  | LIFARA | 1 | 96 | 8 180 |
|  |  | 2 | 109 | 9 304 |
|  |  | 3 | 85 | 7 230 |
|  | REVANSCH | 1 | 102 | 8 726 |
|  |  | 2 | 106 | 9 018 |
|  |  | 3 | 90 | 7 689 |
|  | SW MINTO | 1 | 101 | 8 568 |
|  |  | 2 | 99 | 8 433 |
|  |  | 3 | 94 | 8 048 |
|  | VALTTERI | 1 | 104 | 8 869 |
|  |  | 2 | 109 | 9 266 |
|  |  | 3 | 96 | 8 184 |
| W-THAW | BOR 20203 | 1 | 97 | 8 419 |
|  |  | 2 | 99 | 8 607 |
|  |  | 3 | 115 | 9 999 |
|  | BOR 836 | 1 | 90 | 7 790 |
|  |  | 2 | 92 | 8 021 |
|  |  | 3 | 107 | 9 302 |
|  | FP 6 | 1 | 86 | 7 484 |
|  |  | 2 | 93 | 8 095 |
|  |  | 3 | 114 | 9 935 |
|  | INKERI | 1 | 95 | 8 283 |
|  |  | 2 | 97 | 8 374 |
|  |  | 3 | 110 | 9 505 |
|  | KLAARA | 1 | 96 | 8 307 |
|  |  | 2 | 104 | 9 046 |
|  |  | 3 | 112 | 9 730 |
|  | LIFARA | 1 | 85 | 7 396 |
|  |  | 2 | 92 | 7 995 |
|  |  | 3 | 116 | 10 033 |
|  | REVANSCH | 1 | 94 | 8 130 |
|  |  | 2 | 94 | 8 125 |
|  |  | 3 | 108 | 9 413 |
|  | SW MINTO | 1 | 93 | 8 056 |
|  |  | 2 | 96 | 8 311 |
|  |  | 3 | 110 | 9 569 |
|  | VALTTERI | 1 | 102 | 8 868 |
|  |  | 2 | 92 | 7 959 |
|  |  | 3 | 110 | 9 533 |

Online resources 4. The effects of the tested agro-climatic variables to the yields of tall fescue cultivars.

| Agro-climatic variable | Cultivar | Category | Yield % of average | Mean |
| --- | --- | --- | --- | --- |
| GP-DD5 | KAROLINA | 1 | 90 | 9 093 |
|  |  | 2 | 100 | 10 182 |
|  |  | 3 | 98 | 9 973 |
|  | KORA | 1 | 88 | 8 928 |
|  |  | 2 | 111 | 11 269 |
|  |  | 3 | 109 | 11 084 |
|  | SWAJ | 1 | 89 | 9 033 |
|  |  | 2 | 105 | 10 667 |
|  |  | 3 | 109 | 11 010 |
| GP-TEMP_2_7 | KAROLINA | 1 | 106 | 10 733 |
|  |  | 2 | 96 | 9 720 |
|  |  | 3 | 89 | 8 983 |
|  | KORA | 1 | 110 | 11 150 |
|  |  | 2 | 103 | 10 421 |
|  |  | 3 | 95 | 9 674 |
|  | SWAJ | 1 | 107 | 10 892 |
|  |  | 2 | 100 | 10 133 |
|  |  | 3 | 94 | 9 497 |
| GP-TEMP | KAROLINA | 1 | 100 | 10 191 |
|  |  | 2 | 98 | 9 959 |
|  |  | 3 | 95 | 9 618 |
|  | KORA | 1 | 101 | 10 247 |
|  |  | 2 | 101 | 10 304 |
|  |  | 3 | 104 | 10 598 |
|  | SWAJ | 1 | 98 | 9 970 |
|  |  | 2 | 99 | 10 102 |
|  |  | 3 | 104 | 10 559 |
| GP-RAIN_14 | KAROLINA | 1 | 94 | 9 955 |
|  |  | 2 | 100 | 10 566 |
|  |  | 3 | 98 | 10 305 |
|  | KORA | 1 | 102 | 10 776 |
|  |  | 2 | 105 | 11 040 |
|  |  | 3 | 103 | 10 863 |
|  | SWAJ | 1 | 99 | 10 417 |
|  |  | 2 | 101 | 10 694 |
|  |  | 3 | 99 | 10 417 |
| GP-RAIN_1 | KAROLINA | 1 | 97 | 10 101 |
|  |  | 2 | 94 | 9 842 |
|  |  | 3 | 99 | 10 382 |
|  | KORA | 1 | 102 | 10 652 |
|  |  | 2 | 102 | 10 692 |
|  |  | 3 | 103 | 10 794 |
|  | SWAJ | 1 | 97 | 10 124 |
|  |  | 2 | 105 | 10 965 |
|  |  | 3 | 100 | 10 416 |
| GP-RAIN_2 | KAROLINA | 1 | 96 | 9 998 |
|  |  | 2 | 98 | 10 200 |
|  |  | 3 | 98 | 10 222 |
|  | KORA | 1 | 106 | 11 065 |
|  |  | 2 | 102 | 10 637 |
|  |  | 3 | 101 | 10 473 |
|  | SWAJ | 1 | 99 | 10 349 |
|  |  | 2 | 101 | 10 514 |
|  |  | 3 | 98 | 10 205 |
| GP-TEMP25_1 | KAROLINA | 1 | 96 | 10 040 |
|  |  | 2 | 102 | 10 679 |
|  |  | 3 | 95 | 9 923 |
|  | KORA | 1 | 102 | 10 638 |
|  |  | 2 | 108 | 11 304 |
|  |  | 3 | 96 | 10 011 |
|  | SWAJ | 1 | 98 | 10 278 |
|  |  | 2 | 101 | 10 556 |
|  |  | 3 | 101 | 10 530 |
| GP-TEMP25_2 | KAROLINA | 1 | 99 | 10 151 |
|  |  | 2 | 99 | 10 162 |
|  |  | 3 | 94 | 9 618 |
|  | KORA | 1 | 103 | 10 573 |
|  |  | 2 | 98 | 10 073 |
|  |  | 3 | 103 | 10 565 |
|  | SWAJ | 1 | 99 | 10 165 |
|  |  | 2 | 104 | 10 663 |
|  |  | 3 | 101 | 10 356 |
| GP-TEMP28 | KAROLINA | 1 | 97 | 9 936 |
|  |  | 2 | 97 | 9 926 |
|  |  | 3 | 93 | 9 475 |
|  | KORA | 1 | 99 | 10 081 |
|  |  | 2 | 104 | 10 626 |
|  |  | 3 | 106 | 10 831 |
|  | SWAJ | 1 | 96 | 9 789 |
|  |  | 2 | 105 | 10 711 |
|  |  | 3 | 103 | 10 544 |
| FH | KAROLINA | 1 | 94 | 9 492 |
|  |  | 2 | 102 | 10 312 |
|  |  | 3 | 99 | 9 984 |
|  | KORA | 1 | 102 | 10 260 |
|  |  | 2 | 102 | 10 350 |
|  |  | 3 | 102 | 10 252 |
|  | SWAJ | 1 | 94 | 9 507 |
|  |  | 2 | 101 | 10 234 |
|  |  | 3 | 104 | 10 508 |
| FH-COLD | KAROLINA | 1 | 102 | 10 229 |
|  |  | 2 | 96 | 9 596 |
|  |  | 3 | 94 | 9 469 |
|  | KORA | 1 | 109 | 10 912 |
|  |  | 2 | 99 | 9 975 |
|  |  | 3 | 99 | 9 884 |
|  | SWAJ | 1 | 105 | 10 546 |
|  |  | 2 | 101 | 10 168 |
|  |  | 3 | 94 | 9 458 |
| FH-RAIN | KAROLINA | 1 | 91 | 9 211 |
|  |  | 2 | 97 | 9 736 |
|  |  | 3 | 103 | 10 353 |
|  | KORA | 1 | 99 | 9 954 |
|  |  | 2 | 102 | 10 330 |
|  |  | 3 | 109 | 11 010 |
|  | SWAJ | 1 | 94 | 9 469 |
|  |  | 2 | 103 | 10 402 |
|  |  | 3 | 102 | 10 334 |
| W-STRESS | KAROLINA | 1 | 104 | 10 417 |
|  |  | 2 | 102 | 10 193 |
|  |  | 3 | 85 | 8 544 |
|  | KORA | 1 | 107 | 10 763 |
|  |  | 2 | 110 | 11 028 |
|  |  | 3 | 89 | 8 934 |
|  | SWAJ | 1 | 104 | 10 375 |
|  |  | 2 | 106 | 10 647 |
|  |  | 3 | 93 | 9 305 |
| W-THAW | KAROLINA | 1 | 91 | 9 056 |
|  |  | 2 | 89 | 8 840 |
|  |  | 3 | 110 | 10 983 |
|  | KORA | 1 | 90 | 8 990 |
|  |  | 2 | 100 | 9 971 |
|  |  | 3 | 116 | 11 574 |
|  | SWAJ | 1 | 92 | 9 187 |
|  |  | 2 | 98 | 9 703 |
|  |  | 3 | 113 | 11 193 |

Online resources 5. The effects of the tested agro-climatic variables to the yields of festulolium cultivars.

| Agro-climatic variable | Cultivar | Category | Yield % of average | Mean |
| --- | --- | --- | --- | --- |
| GP-DD5 | FELINA | 1 | 96 | 8 592 |
|  |  | 2 | 111 | 9 949 |
|  |  | 3 | 111 | 9 938 |
|  | FOJTAN | 1 | 62 | 5 551 |
|  |  | 2 | 95 | 8 542 |
|  |  | 3 | 100 | 9 000 |
|  | HYKOR | 1 | 90 | 8 067 |
|  |  | 2 | 118 | 10 610 |
|  |  | 3 | 119 | 10 669 |
| GP-TEMP_2_7 | FELINA | 1 | 109 | 10 362 |
|  |  | 2 | 100 | 9 478 |
|  |  | 3 | 91 | 8 592 |
|  | FOJTAN | 1 | 98 | 9 235 |
|  |  | 2 | 83 | 7 884 |
|  |  | 3 | 103 | 9 731 |
|  | HYKOR | 1 | 112 | 10 601 |
|  |  | 2 | 102 | 9 619 |
|  |  | 3 | 102 | 9 686 |
| GP-TEMP | FELINA | 1 | 103 | 9 788 |
|  |  | 2 | 105 | 9 940 |
|  |  | 3 | 99 | 9 353 |
|  | FOJTAN | 1 | 94 | 8 929 |
|  |  | 2 | 85 | 8 083 |
|  | HYKOR | 1 | 99 | 9 336 |
|  |  | 2 | 110 | 10 376 |
|  |  | 3 | 105 | 9 915 |
| GP-RAIN_14 | FELINA | 1 | 99 | 9 506 |
|  |  | 2 | 104 | 9 982 |
|  |  | 3 | 106 | 10 201 |
|  | FOJTAN | 1 | 90 | 8 630 |
|  |  | 2 | 85 | 8 151 |
|  |  | 3 | 97 | 9 386 |
|  | HYKOR | 1 | 105 | 10 074 |
|  |  | 2 | 109 | 10 486 |
|  |  | 3 | 107 | 10 338 |
| GP-RAIN_1 | FELINA | 1 | 107 | 9 751 |
|  |  | 2 | 108 | 9 915 |
|  |  | 3 | 103 | 9 386 |
|  | FOJTAN | 1 | 97 | 8 883 |
|  |  | 2 | 98 | 8 997 |
|  |  | 3 | 64 | 5 833 |
|  | HYKOR | 1 | 114 | 10 425 |
|  |  | 2 | 105 | 9 568 |
|  |  | 3 | 105 | 9 604 |
| GP-RAIN_2 | FELINA | 1 | 102 | 9 674 |
|  |  | 2 | 108 | 10 162 |
|  |  | 3 | 98 | 9 263 |
|  | FOJTAN | 1 | 86 | 8 109 |
|  |  | 2 | 87 | 8 245 |
|  |  | 3 | 103 | 9 697 |
|  | HYKOR | 1 | 112 | 10 546 |
|  |  | 2 | 110 | 10 427 |
|  |  | 3 | 94 | 8 877 |
| GP-TEMP25_1 | FELINA | 1 | 97 | 9 417 |
|  |  | 2 | 107 | 10 382 |
|  |  | 3 | 101 | 9 816 |
|  | FOJTAN | 1 | 92 | 8 936 |
|  |  | 2 | 84 | 8 181 |
|  | HYKOR | 1 | 98 | 9 532 |
|  |  | 2 | 113 | 10 952 |
|  |  | 3 | 108 | 10 478 |
| GP-TEMP25_2 | FELINA | 1 | 107 | 10 016 |
|  |  | 2 | 95 | 8 951 |
|  |  | 3 | 102 | 9 614 |
|  | FOJTAN | 1 | 93 | 8 769 |
|  |  | 2 | 100 | 9 362 |
|  |  | 3 | 85 | 7 938 |
|  | HYKOR | 1 | 105 | 9 817 |
|  |  | 2 | 104 | 9 804 |
|  |  | 3 | 109 | 10 203 |
| GP-TEMP28 | FELINA | 1 | 100 | 9 397 |
|  |  | 2 | 105 | 9 853 |
|  |  | 3 | 102 | 9 546 |
|  | FOJTAN | 1 | 95 | 8 911 |
|  |  | 2 | 92 | 8 651 |
|  |  | 3 | 85 | 7 927 |
|  | HYKOR | 1 | 98 | 9 142 |
|  |  | 2 | 112 | 10 496 |
|  |  | 3 | 110 | 10 339 |
| FH | FELINA | 1 | 101 | 9 421 |
|  |  | 2 | 106 | 9 885 |
|  |  | 3 | 103 | 9 657 |
|  | FOJTAN | 1 | 96 | 8 927 |
|  |  | 2 | 96 | 8 958 |
|  |  | 3 | 86 | 8 043 |
|  | HYKOR | 1 | 99 | 9 233 |
|  |  | 2 | 104 | 9 750 |
|  |  | 3 | 110 | 10 239 |
| FH-COLD | FELINA | 1 | 109 | 10 069 |
|  |  | 2 | 101 | 9 307 |
|  |  | 3 | 101 | 9 312 |
|  | FOJTAN | 1 | 89 | 8 257 |
|  |  | 2 | 89 | 8 237 |
|  |  | 3 | 97 | 8 976 |
|  | HYKOR | 1 | 115 | 10 575 |
|  |  | 2 | 101 | 9 308 |
|  |  | 3 | 98 | 9 015 |
| FH-RAIN | FELINA | 1 | 96 | 8 882 |
|  |  | 2 | 100 | 9 264 |
|  |  | 3 | 110 | 10 218 |
|  | FOJTAN | 1 | 83 | 7 746 |
|  |  | 2 | 85 | 7 905 |
|  |  | 3 | 106 | 9 901 |
|  | HYKOR | 1 | 104 | 9 679 |
|  |  | 2 | 99 | 9 191 |
|  |  | 3 | 117 | 10 913 |
| W-STRESS | FELINA | 1 | 106 | 9 870 |
|  |  | 2 | 108 | 10 004 |
|  |  | 3 | 96 | 8 920 |
|  | FOJTAN | 1 | 98 | 9 072 |
|  |  | 2 | 101 | 9 416 |
|  |  | 3 | 77 | 7 171 |
|  | HYKOR | 1 | 110 | 10 228 |
|  |  | 2 | 115 | 10 707 |
|  |  | 3 | 89 | 8 231 |
| W-THAW | FELINA | 1 | 92 | 8 376 |
|  |  | 2 | 95 | 8 662 |
|  |  | 3 | 119 | 10 802 |
|  | FOJTAN | 1 | 89 | 8 106 |
|  |  | 2 | 89 | 8 153 |
|  |  | 3 | 101 | 9 197 |
|  | HYKOR | 1 | 95 | 8 643 |
|  |  | 2 | 99 | 9 018 |
|  |  | 3 | 121 | 11 042 |

Online resources 6. The effects of the tested agro-climatic variables to the yields of red clover cultivars.

| Agro-climatic variable | Cultivar | Category | Yield % of average | Mean |
| --- | --- | --- | --- | --- |
| GP-DD5 | PERTTULI | 1 | 82 | 5 474 |
|  |  | 2 | 101 | 6 718 |
|  |  | 3 | 93 | 6 182 |
|  | SUEZ | 1 | 57 | 3 789 |
|  |  | 2 | 100 | 6 642 |
|  |  | 3 | 90 | 6 003 |
|  | SW ARES | 1 | 79 | 5 218 |
|  |  | 2 | 101 | 6 727 |
|  |  | 3 | 105 | 6 983 |
|  | SW TORUN | 1 | 105 | 6 983 |
|  |  | 2 | 131 | 8 702 |
|  |  | 3 | 124 | 8 216 |
|  | SW YNGVE | 1 | 95 | 6 310 |
|  |  | 2 | 120 | 7 943 |
|  |  | 3 | 117 | 7 798 |
| GP-TEMP_2_7 | PERTTULI | 1 | 106 | 7 185 |
|  |  | 2 | 79 | 5 352 |
|  |  | 3 | 91 | 6 157 |
|  | SUEZ | 2 | 90 | 6 084 |
|  |  | 3 | 85 | 5 795 |
|  | SW ARES | 2 | 97 | 6 554 |
|  |  | 3 | 97 | 6 555 |
|  | SW TORUN | 1 | 120 | 8 134 |
|  |  | 2 | 96 | 6 494 |
|  |  | 3 | 122 | 8 257 |
|  | SW YNGVE | 2 | 108 | 7 303 |
|  |  | 3 | 111 | 7 505 |
| GP-TEMP | PERTTULI | 1 | 72 | 4 962 |
|  |  | 2 | 92 | 6 352 |
|  |  | 3 | 101 | 6 932 |
|  | SUEZ | 2 | 80 | 5 466 |
|  |  | 3 | 95 | 6 494 |
|  | SW ARES | 2 | 96 | 6 609 |
|  |  | 3 | 104 | 7 141 |
|  | SW TORUN | 1 | 87 | 5 976 |
|  |  | 2 | 120 | 8 245 |
|  |  | 3 | 127 | 8 724 |
|  | SW YNGVE | 2 | 108 | 7 418 |
|  |  | 3 | 119 | 8 149 |
| GP-RAIN_14 | PERTTULI | 1 | 91 | 6 251 |
|  |  | 2 | 86 | 5 880 |
|  |  | 3 | 100 | 6 860 |
|  | SUEZ | 1 | 82 | 5 599 |
|  |  | 2 | 76 | 5 186 |
|  |  | 3 | 97 | 6 612 |
|  | SW ARES | 1 | 100 | 6 867 |
|  |  | 2 | 88 | 6 004 |
|  |  | 3 | 100 | 6 835 |
|  | SW TORUN | 1 | 113 | 7 768 |
|  |  | 2 | 114 | 7 794 |
|  |  | 3 | 125 | 8 558 |
|  | SW YNGVE | 1 | 112 | 7 691 |
|  |  | 2 | 107 | 7 299 |
|  |  | 3 | 110 | 7 530 |
| GP-RAIN_1 | PERTTULI | 1 | 90 | 6 159 |
|  |  | 2 | 107 | 7 333 |
|  |  | 3 | 87 | 5 950 |
|  | SUEZ | 1 | 88 | 6 002 |
|  |  | 2 | 77 | 5 266 |
|  |  | 3 | 85 | 5 805 |
|  | SW ARES | 1 | 83 | 5 722 |
|  |  | 2 | 104 | 7 130 |
|  |  | 3 | 93 | 6 377 |
|  | SW TORUN | 1 | 121 | 8 266 |
|  |  | 2 | 123 | 8 428 |
|  |  | 3 | 114 | 7 821 |
|  | SW YNGVE | 1 | 106 | 7 270 |
|  |  | 2 | 118 | 8 065 |
|  |  | 3 | 106 | 7 245 |
| GP-RAIN_2 | PERTTULI | 1 | 83 | 5 640 |
|  |  | 2 | 91 | 6 180 |
|  |  | 3 | 98 | 6 677 |
|  | SUEZ | 1 | 86 | 5 837 |
|  |  | 2 | 81 | 5 492 |
|  |  | 3 | 89 | 6 042 |
|  | SW ARES | 1 | 106 | 7 202 |
|  |  | 2 | 91 | 6 159 |
|  |  | 3 | 98 | 6 656 |
|  | SW TORUN | 1 | 107 | 7 249 |
|  |  | 2 | 115 | 7 810 |
|  |  | 3 | 125 | 8 502 |
|  | SW YNGVE | 1 | 112 | 7 600 |
|  |  | 2 | 108 | 7 330 |
|  |  | 3 | 112 | 7 646 |
| GP-TEMP25_1 | PERTTULI | 1 | 92 | 6 069 |
|  |  | 2 | 94 | 6 213 |
|  |  | 3 | 94 | 6 232 |
|  | SUEZ | 1 | 87 | 5 735 |
|  |  | 2 | 73 | 4 832 |
|  |  | 3 | 90 | 5 940 |
|  | SW ARES | 1 | 92 | 6 105 |
|  |  | 2 | 86 | 5 687 |
|  |  | 3 | 103 | 6 814 |
|  | SW TORUN | 1 | 121 | 7 997 |
|  |  | 2 | 115 | 7 656 |
|  |  | 3 | 121 | 7 994 |
|  | SW YNGVE | 1 | 102 | 6 787 |
|  |  | 2 | 116 | 7 711 |
|  |  | 3 | 116 | 7 664 |
| GP-TEMP25_2 | PERTTULI | 1 | 89 | 5 955 |
|  |  | 2 | 93 | 6 209 |
|  |  | 3 | 97 | 6 473 |
|  | SUEZ | 1 | 55 | 3 674 |
|  |  | 2 | 97 | 6 490 |
|  |  | 3 | 93 | 6 215 |
|  | SW ARES | 1 | 78 | 5 224 |
|  |  | 2 | 100 | 6 684 |
|  |  | 3 | 105 | 7 049 |
|  | SW TORUN | 1 | 111 | 7 408 |
|  |  | 2 | 124 | 8 289 |
|  |  | 3 | 125 | 8 375 |
|  | SW YNGVE | 1 | 106 | 7 097 |
|  |  | 2 | 108 | 7 208 |
|  |  | 3 | 119 | 7 984 |
| GP-TEMP28 | PERTTULI | 1 | 89 | 5 976 |
|  |  | 2 | 102 | 6 855 |
|  |  | 3 | 83 | 5 558 |
|  | SUEZ | 1 | 69 | 4 592 |
|  |  | 2 | 114 | 7 621 |
|  |  | 3 | 75 | 5 050 |
|  | SW ARES | 1 | 90 | 6 051 |
|  |  | 2 | 105 | 7 032 |
|  |  | 3 | 89 | 5 942 |
|  | SW TORUN | 1 | 110 | 7 340 |
|  |  | 2 | 135 | 9 067 |
|  |  | 3 | 110 | 7 393 |
|  | SW YNGVE | 1 | 102 | 6 808 |
|  |  | 2 | 124 | 8 273 |
|  |  | 3 | 103 | 6 893 |
| FH | PERTTULI | 1 | 87 | 6 055 |
|  |  | 2 | 93 | 6 492 |
|  |  | 3 | 96 | 6 724 |
|  | SUEZ | 1 | 103 | 7 170 |
|  |  | 2 | 74 | 5 150 |
|  |  | 3 | 90 | 6 256 |
|  | SW ARES | 1 | 85 | 5 931 |
|  |  | 2 | 94 | 6 543 |
|  |  | 3 | 98 | 6 875 |
|  | SW TORUN | 1 | 108 | 7 554 |
|  |  | 2 | 114 | 7 962 |
|  |  | 3 | 124 | 8 682 |
|  | SW YNGVE | 1 | 112 | 7 826 |
|  |  | 2 | 114 | 7 991 |
|  |  | 3 | 107 | 7 482 |
| FH-COLD | PERTTULI | 1 | 86 | 5 910 |
|  |  | 2 | 101 | 6 890 |
|  |  | 3 | 95 | 6 481 |
|  | SUEZ | 1 | 85 | 5 793 |
|  |  | 2 | 83 | 5 669 |
|  |  | 3 | 80 | 5 497 |
|  | SW ARES | 1 | 94 | 6 431 |
|  |  | 2 | 94 | 6 426 |
|  |  | 3 | 98 | 6 684 |
|  | SW TORUN | 1 | 115 | 7 913 |
|  |  | 2 | 116 | 7 950 |
|  |  | 3 | 122 | 8 341 |
|  | SW YNGVE | 1 | 102 | 6 984 |
|  |  | 2 | 111 | 7 574 |
|  |  | 3 | 120 | 8 223 |
| FH-RAIN | PERTTULI | 1 | 96 | 6 829 |
|  |  | 2 | 91 | 6 444 |
|  |  | 3 | 93 | 6 630 |
|  | SUEZ | 1 | 86 | 6 124 |
|  |  | 2 | 80 | 5 687 |
|  |  | 3 | 83 | 5 912 |
|  | SW ARES | 1 | 92 | 6 558 |
|  |  | 2 | 93 | 6 592 |
|  |  | 3 | 106 | 7 569 |
|  | SW TORUN | 1 | 115 | 8 167 |
|  |  | 2 | 116 | 8 259 |
|  |  | 3 | 128 | 9 064 |
|  | SW YNGVE | 1 | 104 | 7 424 |
|  |  | 2 | 111 | 7 923 |
|  |  | 3 | 105 | 7 443 |
| W-STRESS | PERTTULI | 1 | 96 | 6 551 |
|  |  | 2 | 85 | 5 821 |
|  |  | 3 | 96 | 6 542 |
|  | SUEZ | 1 | 88 | 6 014 |
|  |  | 2 | 76 | 5 186 |
|  |  | 3 | 90 | 6 126 |
|  | SW ARES | 1 | 104 | 7 085 |
|  |  | 2 | 86 | 5 857 |
|  |  | 3 | 101 | 6 902 |
|  | SW TORUN | 1 | 118 | 8 078 |
|  |  | 2 | 117 | 8 001 |
|  |  | 3 | 121 | 8 227 |
|  | SW YNGVE | 1 | 100 | 6 793 |
|  |  | 2 | 106 | 7 220 |
|  |  | 3 | 116 | 7 937 |
| W-THAW | PERTTULI | 1 | 84 | 5 640 |
|  |  | 2 | 112 | 7 526 |
|  |  | 3 | 89 | 5 956 |
|  | SUEZ | 1 | 54 | 3 620 |
|  |  | 2 | 100 | 6 708 |
|  |  | 3 | 85 | 5 734 |
|  | SW ARES | 1 | 83 | 5 576 |
|  |  | 2 | 112 | 7 524 |
|  |  | 3 | 91 | 6 122 |
|  | SW TORUN | 1 | 106 | 7 140 |
|  |  | 2 | 140 | 9 405 |
|  |  | 3 | 113 | 7 584 |
|  | SW YNGVE | 1 | 101 | 6 798 |
|  |  | 2 | 123 | 8 234 |
|  |  | 3 | 106 | 7 104 |
